# Dengue virus nonstructural 3 protein interacts directly with human glyceraldehyde-3-phosphate dehydrogenase (GAPDH) and reduces its glycolytic activity

**DOI:** 10.1038/s41598-019-39157-7

**Published:** 2019-02-25

**Authors:** Emiliana M. Silva, Jonas N. Conde, Diego Allonso, Gustavo T. Ventura, Diego R. Coelho, Pedro Henrique Carneiro, Manuela L. Silva, Marciano V. Paes, Kíssila Rabelo, Gilberto Weissmuller, Paulo Mascarello Bisch, Ronaldo Mohana-Borges

**Affiliations:** 1 Laboratório de Genômica Estrutural, Instituto de Biofísica Carlos Chagas Filho, Universidade Federal do Rio de Janeiro, Rio de Janeiro, RJ 21941-590 Brazil; 2 Departamento de Biotecnologia Farmacêutica, Faculdade de Farmácia, Universidade Federal do Rio de Janeiro, Rio de Janeiro, RJ Brazil; 3 Instituto de Biodiversidade e Sustentabilidade (NUPEM/UFRJ), Universidade Federal do Rio de Janeiro, Macaé, RJ Brazil; 4 Laboratório Interdisciplinar de Pesquisa Médica, Instituto Oswaldo Cruz, Fundação Oswaldo Cruz, Rio de Janeiro, RJ Brazil; 5 Laboratório de Ultraestrutura e Biologia Tecidual, Universidade Estadual do Rio de Janeiro, Rio de Janeiro, RJ Brazil; 6 Laboratório de Física Biológica, Instituto de Biofísica Carlos Chagas Filho, Universidade Federal do Rio de Janeiro, Rio de Janeiro, RJ 21941-590 Brazil

## Abstract

Dengue is an important mosquito-borne disease and a global public health problem. The disease is caused by dengue virus (DENV), which is a member of the Flaviviridae family and contains a positive single-stranded RNA genome that encodes a single precursor polyprotein that is further cleaved into structural and non-structural proteins. Among these proteins, the non-structural 3 (NS3) protein is very important because it forms a non-covalent complex with the NS2B cofactor, thereby forming the functional viral protease. NS3 also contains a C-terminal ATPase/helicase domain that is essential for RNA replication. Here, we identified 47 NS3-interacting partners using the yeast two-hybrid system. Among those partners, we highlight several proteins involved in host energy metabolism, such as apolipoprotein H, aldolase B, cytochrome C oxidase and glyceraldehyde-3-phosphate dehydrogenase (GAPDH). GAPDH directly binds full-length NS3 and its isolated helicase and protease domains. Moreover, we observed an intense colocalization between the GAPDH and NS3 proteins in DENV2-infected Huh7.5.1 cells, in NS3-transfected BHK-21 cells and in hepatic tissue from a fatal dengue case. Taken together, these results suggest that the human GAPDH-DENV NS3 interaction is involved in hepatic metabolic alterations, which may contribute to the appearance of steatosis in dengue-infected patients. The interaction between GAPDH and full-length NS3 or its helicase domain *in vitro* as well as in NS3-transfected cells resulted in decreased GAPDH glycolytic activity. Reduced GAPDH glycolytic activity may lead to the accumulation of metabolic intermediates, shifting metabolism to alternative, non-glycolytic pathways. This report is the first to identify the interaction of the DENV2 NS3 protein with the GAPDH protein and to demonstrate that this interaction may play an important role in the molecular mechanism that triggers hepatic alterations.

## Introduction

Dengue virus (DENV) belongs to the Flaviviridae family, which also includes 70 other viruses, such as yellow fever virus (YFV), Zika virus, Japanese encephalitis virus (JEV) and West Nile virus^1^. Currently, four distinct DENV serotypes (DENV1 to 4) are transmitted to humans by *Aedes* mosquitos^2–4^, and consecutive infections with different DENV serotypes are commonly associated with severe outcomes^5^. The absence of an adequate experimental animal model has hampered major scientific progress regarding dengue pathogenesis and consequently the development of therapeutics, preventing the control of the disease and resulting in frequent dengue outbreaks worldwide^6,7^.

A dengue vaccine has been commercialized only recently. The chimeric yellow fever-DENV tetravalent dengue vaccine (CYD-TDV) is a live-attenuated vaccine that expresses the structural antigens of the four DENV serotypes, the membrane protein (prM) and envelope protein (E), which act as targets for the host immune response^8,9^. However, several factors, such as age, host physiology and repeated exposure to DENV, have been observed to affect vaccine efficacy^9^. The company claims a vaccine efficacy of approximately 65% against DENV2^9^.

Recent estimates indicate that approximately 390 million dengue infections occur annually^10^. DENV infections can range from asymptomatic cases to life-threatening hypovolemic shock^1^. The molecular mechanisms underlying severe disease remain under discussion. However, immunopathological studies have demonstrated that DENV tropism for immune, liver, lung and endothelial cells is responsible for irreversible organ injury, which has been observed in dengue hemorrhagic fever (DHF) and dengue shock syndrome (DSS) pathogenesis^11,12^.

DENV is an enveloped virus that contains a nucleocapsid composed of a capsid protein (C) and a positive single-stranded RNA molecule^4^, which encodes a unique polyprotein that is processed by cellular and viral proteases into three structural proteins (C, prM/M, and E) and seven non-structural proteins (NS1, NS2A, NS2B, NS3, NS4A, NS4B, and NS5)^2^. The non-structural proteins are known to be directly involved in viral replication and assembly^4,13^.

NS3 is a highly conserved protein among flaviviruses. The NS3 N-terminal region contains a protease catalytic domain that forms a non-covalent complex with the NS2B cofactor for its optimum proteolytic activity. The NS2B protein is located upstream the NS3 protease domain and functions as a cofactor by promoting important conformational changes in the NS3 structure^14^. Previous studies showed that the expression of the central conserved 40-amino acid hydrophilic domain of NS2B (CF40) fused to NS3pro was sufficient for efficient cofactor activity^15^. NS2B/NS3 complex is responsible for the proteolytic processing of the viral polyprotein at the NS2A/NS2B, NS2B/NS3, NS3/NS4A and NS4B/NS5 junctions^16^. NS3 also contains an ATPase/helicase and RNA triphosphatase domain in its C-terminal region, which is essential for viral RNA replication and capping^17,18^. Because of its ability to cleave different parts of the polyprotein precursor and its participation in viral replication, NS3 is considered an important target for screening drug candidates and evaluating their efficacy.

Although substantial advances have been made in determining the structure of DENV proteins^19–22^ and their interactions with cellular proteins, our understanding of the mechanisms that control disease severity is far from ideal. Because of its compact genome, DENV probably requires an extensive number of interactions with host factors for its replication^23^. Heaton and colleagues showed that the DENV NS3 protein interacts with fatty acid synthase (FASN) on the endoplasmic reticulum (ER) membrane, where the viral replicative complex assembles, and increases fatty acid biosynthesis during DENV infection^24^.

In the present study, we employed the yeast two-hybrid (Y2H) system to identify putative cellular interacting partners of the DENV2 NS3 protein. Among the 47 NS3-interacting partners, we found the glyceraldehyde-3-phosphate dehydrogenase (GAPDH) enzyme, which catalyzes the oxidative phosphorylation of glyceraldehyde-3-phosphate to 1,3-biphosphoglycerate, leading to the reduction of nicotinamide adenine dinucleotide (NAD^+^) to NADH. This enzyme is a 335-amino-acid polypeptide that has been highly conserved throughout evolution. Although it is translated as a single 37-kDa protein, GAPDH is normally found as a homotetramer with two major domains, the NAD^+^-binding domain (amino acids 1–150) and the catalytic domain that contains the active cysteine (Cys152)^25,26^. GAPDH displays a variety of additional and unrelated activities in addition to its classical glycolytic activity. For example, GAPDH acts as an AU 3′-UTR binding protein via the RNA-binding domain within the NAD^+^ binding site, which is responsible for mRNA stability and translation^27–29^. Recent studies have also shown that interactions with the active site are important for the roles of GAPDH in heme metabolism^30^, in the cellular response to oxidative stress and in apoptosis^31^.

In this study, we demonstrate that human GAPDH interacted with full-length NS3, as well as the isolated NS3 helicase and protease domains, based on ELISA assays, surface plasmon resonance (SPR) measurements and molecular docking. The interaction of both full-length NS3 and its helicase domain with GAPDH resulted in a decrease in the glycolytic activity of GAPDH. The reduction of intracellular GAPDH activity was also detected in both NS3-transfected and DENV2 infected BHK-21 cells, highlighting the importance of this interaction. Moreover, we observed in a coimmunoprecipitation assay that DENV2 NS3 and the human GAPDH protein interacted in DENV2-infected human hepatocyte cells and NS3-transfected cells, and confocal microscopy showed that these proteins colocalized *in vitro*. NS3-transfected cells also supported these results. This is the first report to identify the interaction of full-length DENV2 NS3 and its domains with the GAPDH protein and to demonstrate that this interaction may play an important role in the molecular mechanism that triggers DENV-promoted steatosis.

## Results

### Identification of DENV2 NS3-interacting partners using a yeast two-hybrid system

**Figure 1:**
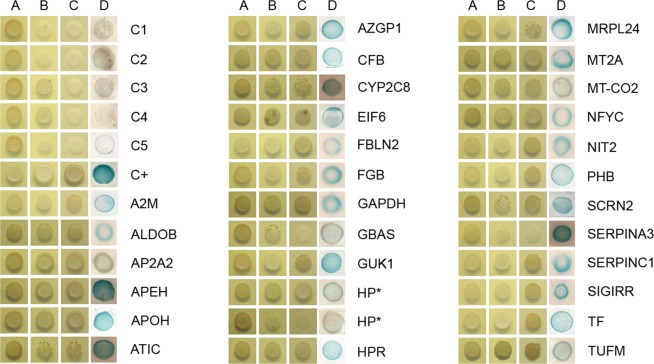
The DENV2 NS3 protein interacts with human liver proteins in the yeast two-hybrid system. The yeast strain AH109 was co-transformed with the NS3 bait plasmid, as well as the prey or control plasmids. The growth of the transformant colonies was visualized on double drop-out medium (SD-Leu-Trp; column **A**). Positive interaction partners were visualized by growth on triple drop-out medium (SD-His-Leu-Trp; column **B**) and quadruple drop-out medium (SD-Ade-His-Leu-Trp; column **C**). Strong interactions were indicated by β-galactosidase expression, as evidenced by blue staining on the colony-lift filter assay (column **D**), indicating HIS3, ADE2 and lacZ reporter gene activation. Transformants containing the plasmids pGBKT7-NS3 and PGADT7-AD (C1), pGBKT7-NS3 and PGADT7-T (SV40 large T-antigen fused to the GAL4 activation domain) (C2), empty pGBKT7 and PGADT7-AD (C3), empty pGBKT7 and PGADT7-T (C4), and pGBKT7-Lam (laminin C) and PGADT7-AD (C5) were used as negative controls. Transformants containing the plasmids pGBKT7-p53 (murine p53 fused to the GAL4 DNA-binding domain) and PGADT7-T were used as positive controls (C+).

As described in the literature, the liver is the solid organ that is most affected by DENV^11^. Liver involvement is usually monitored by elevated hepatic transaminase levels, necrosis and lipid droplet formation within hepatocytes that exhibit macrosteatosis, in which the NS3 protein is also detected^12^. Despite our current understanding of liver involvement during dengue infection, the factors that promote liver injury are poorly understood. Thus, we performed a yeast two-hybrid screen using a human liver cDNA library (prey) to identify novel host factors that are able to interact with the DENV2 NS3 protein (bait). To select putative positive clones, transformed cells containing the bait and prey plasmids were grown in triple drop-out media, resulting in the detection of 2,600 colonies. Forty-seven putative NS3-interacting partners were selected by HIS3, ADE2 and lacZ reporter gene activation, as visualized by colony growth on triple (SD-His-Leu-Trp) and quadruple (SD-Ade-His-Leu-Trp) drop-out plates and by blue staining following colony-lift filter assays to detect β-galactosidase expression (Fig. 1). The isolated prey plasmids were sequenced and analyzed with BLASTX (available on the NCBI website) to identify the corresponding genes that encode the interacting proteins.

Among the NS3-interacting partners, we observed that the majority of the identified proteins are involved in essential metabolic processes, such as purine biosynthesis (5-aminoimidazole-4-carboxamide ribonucleotide formyltransferase), transcription regulation (nuclear transcription factor Y and prohibitin), serine protease activity inhibition (alpha-2-macroglobulin), lipid metabolism regulation (apolipoprotein H), oxidative phosphorylation (cytochrome C oxidase) and glycolysis (aldolase B and glyceraldehyde-3-phosphate dehydrogenase) (Table 1).

**Table 1:** DENV2 NS3-interacting partners identified by the yeast two-hybrid screen.

Abbreviation	Gene Name	NCBI ID	Numbers of clones	Biological Process
A2M	alpha-2-macroglobulin	NM_000014.4	1	Serine Protease Inhibitor
ALDOB	aldolase B, fructose-biphosphate	NM_000035.3	1	Glycolytic Process
AP2A2	adaptor-related protein complex 2, alpha 2 subunit	NM_012305.3	1	Protein Transporter Activity
APEH	acylaminoacyl-peptide hydrolase	NM_001640.3	1	Serine-type Endopeptidase Activity
APOH	apolipoprotein H (beta-2-glycoprotein I)	NM_000042.2	11	Triglyceride Metabolic Process
ATIC	5-aminoimidazole-4-carboxamide ribonucleotide formyltransferase	NM_004044.6	1	Purine Biosynthesis
AZGP1	alpha-2-glycoprotein 1, zinc-binding	NM_001185.3	4	Antigen Binding Ribonuclease Activity
CFB	complement factor B	NM_001710.5	1	Regulation of Complement Activation
CYP2C8	cytochrome P450, family 2, subfamily C, polypeptide 8	NM_000770.3	1	arachidonic acid metabolic process omega-hydroxylase P450 pathway oxidation-reduction process
EIF6	eukaryotic translation initiation factor 6	NM_002212.3	1	translation initiation factor activity
FBLN2	fibulin 2	NM_001998.2	1	extracellular matrix organization
FGB	fibrinogen beta chain	NM_005141.4	2	blood coagulation platelet activation
GAPDH	glyceraldehyde-3-phosphate dehydrogenase	NM_002046.5	1	Glycolytic Process
GBAS	glioblastoma amplified sequence	NM_001483.2	1	ATP biosynthetic process oxidative phosphorylation
GUK1	guanylate kinase 1	NM_001159390.1	1	ATP metabolic process dGDP biosynthetic process
HPR	haptoglobin-related protein	NM_020995.3	5	receptor-mediated endocytosis hemoglobin binding
MRPL24	mitochondrial ribosomal protein L24	NM_024540.3	1	structural constituent of ribosome
MT2A	metallothionein-2A	NM_005953.3	1	cellular copper ion homeostasis
MT-CO2	cytochrome c oxidase subunit II		1	respiratory electron transport chain
NFYC	nuclear transcription factor Y, gamma	NM_001142588.1	1	transcription coactivator activity
NIT2	nitrilase family, member 2	NM_020202.4	1	asparagine, glutamine and oxaloacetate metabolic process
PHB	prohibitin	NM_002634.3	1	histone deacetylase binding negative regulation of cell growth negative regulation of transcription,
SCRN2	secernin 2	NM_138355.3	2	dipeptidase activity exocytosis
SERPINA3	serpin peptidase inhibitor, clade A member 3	NM_001085.4	1	serine-type endopeptidase inhibitor activity regulation of lipid metabolic process
SERPINC1	serpin peptidase inhibitor, clade C (antithrombin), member 1	NM_000488.3	1	serine-type endopeptidase inhibitor activity
SIGIRR	single immunoglobulin and toll-interleukin 1 receptor (TIR) domain	NM_021805.2	1	negative regulation of cytokine-mediated signaling pathway negative regulation of lipopolysaccharide-mediated signaling pathway
TF	transferrin	NM_001063.3	1	blood coagulation
TUFM	Tu translation elongation factor, mitochondrial	NM_003321.4	1	mitochondrial translation

### Human GAPDH interacts directly with DENV2 NS3

**Figure 2:**
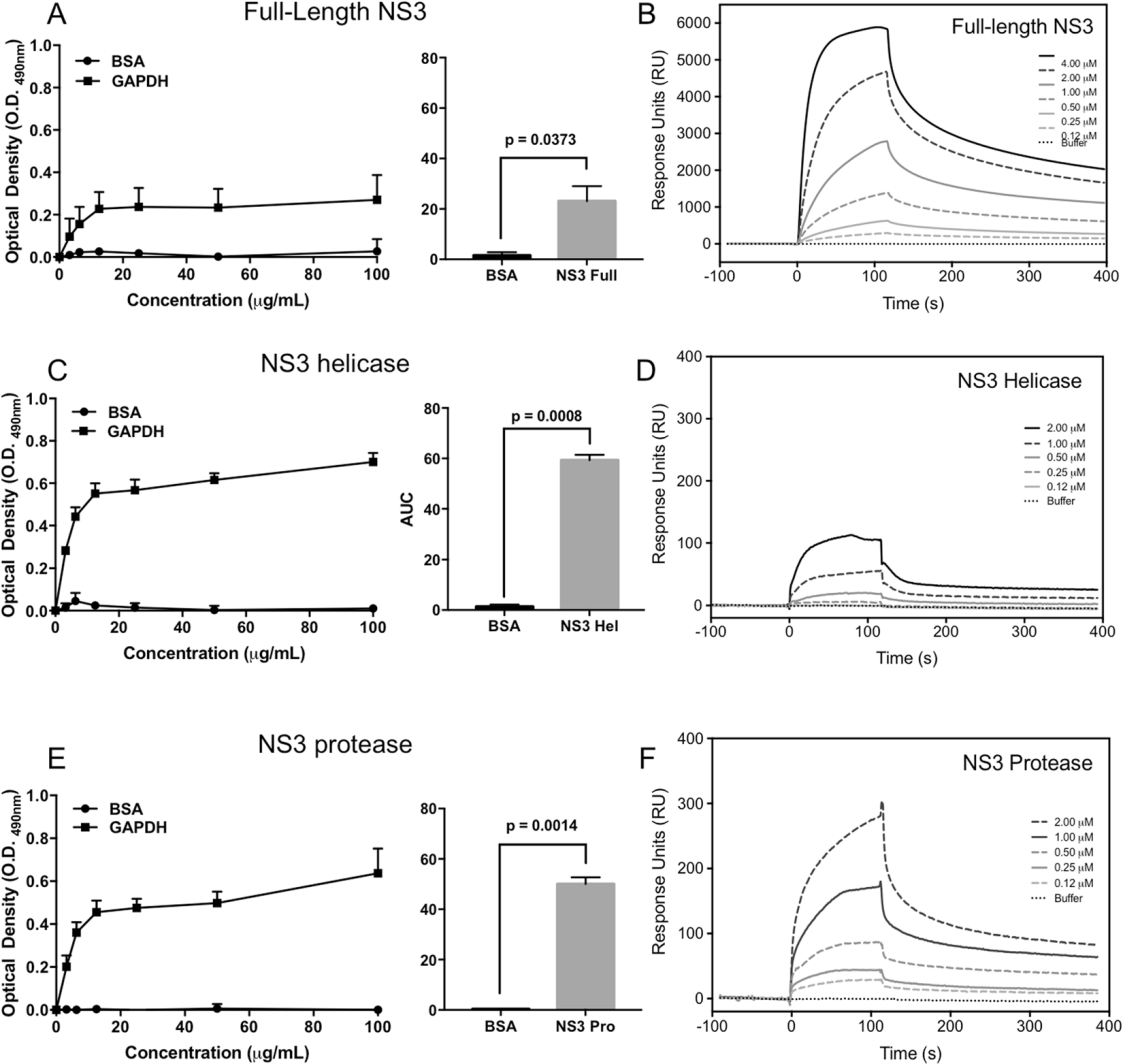
The human GAPDH protein directly binds the full-length DENV2 NS3 protein and its helicase and protease domains. A binding ELISA was performed using microtiter plates coated with 50 μg/mL each of the purified full-length NS3 protein (**A**), NS3 helicase domain (**C**) or NS3 protease domain (**E**), followed by incubation with increasing concentrations of human GAPDH and detection with an anti-GAPDH polyclonal antibody. A two-way ANOVA followed by Bonferroni post-test was used to calculate statistical significance between the control and NS3-containing curves. The bar graphs represent the area under the curve (AUC) for each NS3 construct compared to its corresponding negative control (BSA). The error bars indicate the standard deviation from three independent experiments, and the *p* values show significant differences between NS3 constructs and BSA curves, calculated by unpaired Student’s *t* test. SPR sensorgrams reflect the binding of the recombinant full-length NS3 protein (**B**) and the helicase (**D**) and protease (**F**) domains to the immobilized GAPDH protein. The amount of full-length NS3 and its domains that associated with GAPDH was measured in response units (RU). The global equilibrium dissociation constant values (K_D_) were calculated.

To confirm the interaction between DENV2 NS3 and GAPDH *in vitro*, ELISA assays were performed to evaluate whether GAPDH directly binds the DENV2 NS3 protein by ELISA assays (Fig. 2). ELISA assays were carried out by using 5 µg of each purified protein (full-length NS3 and its helicase and protease domains) or 5 µg of BSA (control) immobilized onto the plates and incubated with increasing concentrations (from 1.6 µg/mL to 100 µg/mL) of purified recombinant human GAPDH protein. A polyclonal rabbit anti-GAPDH antibody was used to detect positive interactions. An increase in the optical density (O.D._490nm_) was observed when the proteins were incubated together, indicating a significant dose-dependent binding of GAPDH to full-length NS3 (Fig. 2A) and its helicase (Fig. 2C) and protease (Fig. 2E) domains. No change in O.D._490nm_ measurements was observed with BSA. Statistical analysis using two-way ANOVA followed by Bonferroni post-test revealed a significant difference between the binding curves and BSA (negative control), represented by the area under the curve (AUC) in the bar graphs.

SPR experiments were also performed to confirm the DENV2 NS3-GAPDH interaction. Human GAPDH was immobilized on a CM5 sensor chip surface and then subjected to injections containing full-length NS3, as well as its helicase and protease domains, at concentrations ranging from 0.06 µM to 4.0 µM, and the bound proteins were measured in arbitrary response units (RU). We observed a concentration-dependent binding of full-length NS3 and its helicase and protease domains to immobilized GAPDH, as shown in Fig. 2B,D,F, respectively. A comparison of the global dissociation equilibrium constants indicated that the affinity of GAPDH for full-length NS3 was higher than that observed for its helicase and protease domains (0.31 µM, 2.5 µM and 2.4 µM, respectively). These results confirm that human GAPDH directly binds DENV2 NS3, as previously demonstrated by the yeast two-hybrid experiment.

### GAPDH glycolytic activity is suppressed by the interaction with the DENV NS3 protein

Glucose uptake is required for the development of DENV infection and the support of viral replication^32,33^. Therefore, we evaluated the effect of the DENV2 NS3 protein on GAPDH glycolytic activity. GAPDH catalyzes a reversible oxidoreductase reaction in which glyceraldehyde-3-phosphate is converted into 1,3-biphosphoglycerate in the presence of nicotinamide ADP (NAD^+^) and inorganic phosphate (P_i_), with the release of NADH. Enzymatic activity was measured after the addition of different concentrations of the full-length NS3 protein or its separate domains (1 or 3 μM) or following the addition of 3 μM BSA (negative control), in reactions containing recombinant human GAPDH protein, β-NADH, ATP, phosphoglycerate kinase (PGK) and 3-phosphoglycerate (PG). β-NAD^+^ production was monitored by the decrease in fluorescence intensity. The relative fluorescence intensity (F/Fo) of β-NAD^+^ production was measured, and the percentage of GAPDH activity was calculated and compared with the values obtained for GAPDH reactions without NS3 proteins or with BSA.

**Figure 3:**
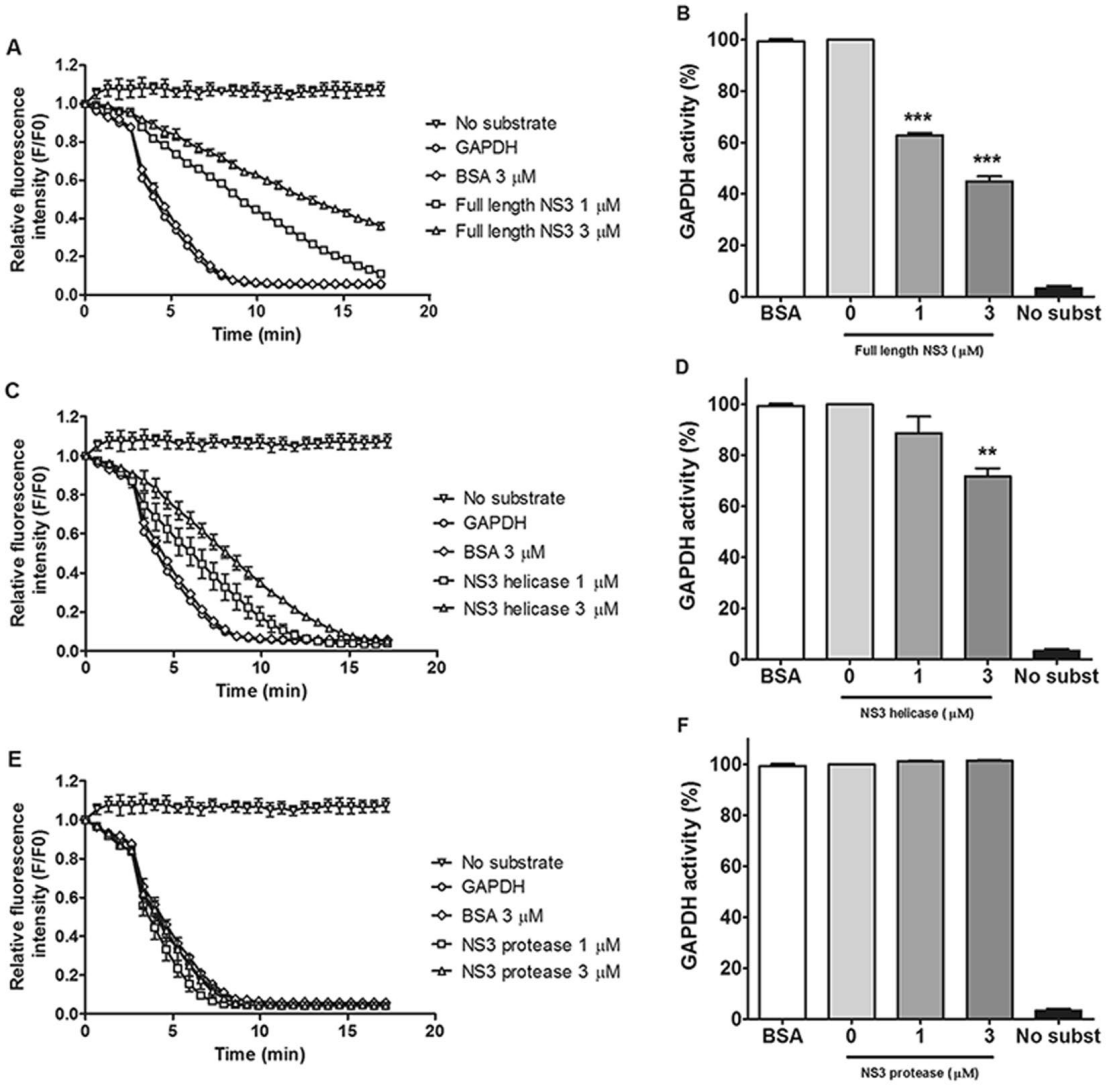
The interaction between GAPDH and recombinant full-length NS3 or the helicase domain decreases GAPDH activity. Enzymatic activity was measured in a buffer containing 50 mM Tris-HCl, pH 7.4, 2 mM MgCl_2_, 1 mM ATP, 1 mM EDTA, 0.25 mM β-NADH, 13 units/mL phosphoglycerate kinase (PGK) and 1 µM human GAPDH. The reaction was incubated with increasing concentrations (1 to 3 µM) of the full-length NS3 protein (**A**,**B**), as well as the helicase (**C**,**D**) and NS3 protease (**E**,**F**) domains. The negative control was performed with 3 µM BSA. The reactions were triggered by the addition of 5 mM 3-phosphoglycerate. GAPDH activity was expressed as the decrease in the relative fluorescence intensity (F/F_0_) of NADH after its conversion to NAD^+^. The relative fluorescence intensity data were then converted to percentages using a representative time point from each reaction (10 min) to better show the effects on GAPDH activity as a function of incubation with different amounts of the NS3 proteins. The data were obtained in three independent experiments, and the bars indicate the standard errors. Statistically significant differences were analyzed using a two-tailed, unpaired Student’s *t-*test. *p < 0.05, **p < 0.01, and ***p < 0.001.

We observed that GAPDH activity was reduced by half of its initial value when 3 μM full-length NS3 protein was added compared with the reactions without NS3 proteins and with 3 μM BSA (Fig. 3A,B). Furthermore, the conversion of β-NADH to β-NAD^+^ was delayed in the presence of the NS3 helicase domain, which promoted an almost 30% decrease in GAPDH glycolytic activity (Fig. 3C,D). No effect on GAPDH activity was observed after the addition of 1 or 3 μM protease domain (Fig. 3E,F). Thus, we concluded that both the full-length NS3 protein and the helicase domain decrease GAPDH activity. Conversely, the protease domain does not modulate the activity of GAPDH.

### ATPase activity of DENV NS3 is increased by GAPDH binding

**Figure 4:**
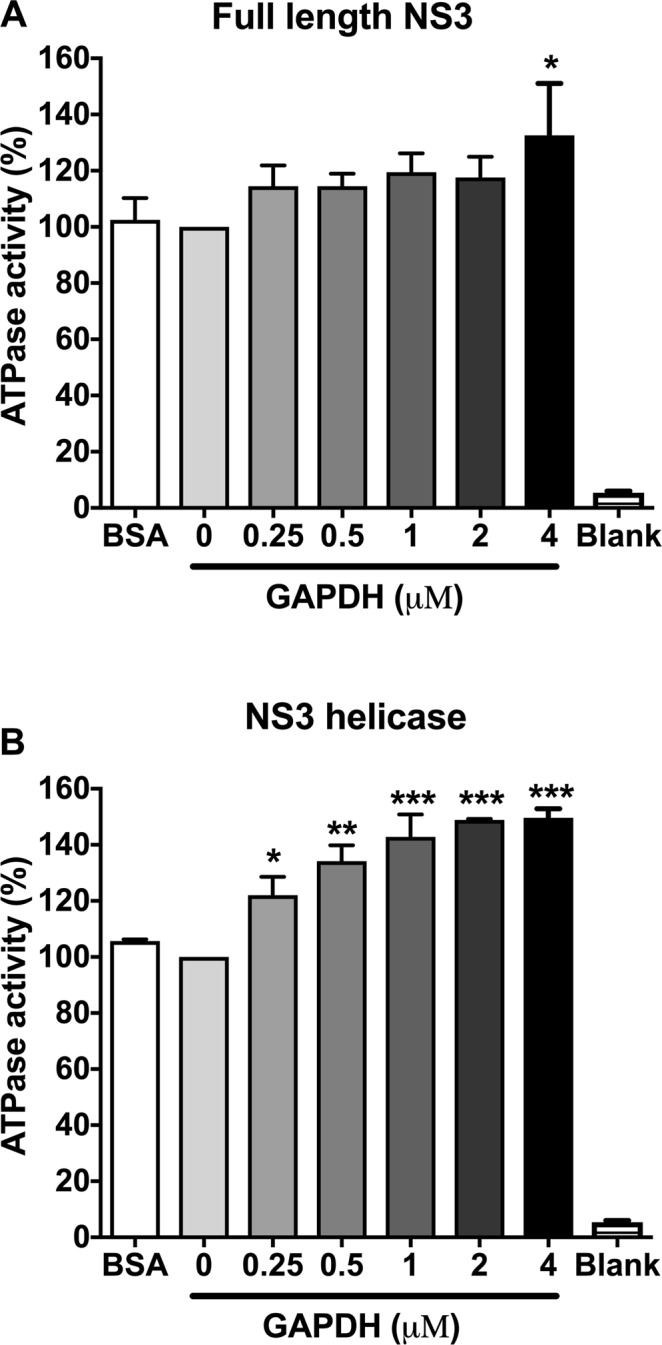
Human GAPDH increases NS3 ATPase activity. The human GAPDH protein (from 0.25 μM to 4 μM) was added to 40-µL reactions containing 40 mM MES-Tris-HCl, pH 7.5, 5 mM DTT, 5 mM MgCl_2,_ 100 mM KCl and 0.3 µM recombinant full-length NS3 protein (**A**) or its helicase domain (**B**). The reactions were triggered with 1 mM ATP. The formation of free Pi resulted in a blue-colored reaction, which was measured at 660 nm after the addition of ammonium molybdate and a reducing agent. The percentage of ATPase activity was calculated relative to the reaction without the GAPDH protein. The negative control was performed with 4 µM BSA. The error bars indicate the standard deviations from three independent experiments, and asterisks indicate significant differences from the control using two-way ANOVA and the Bonferroni post-test. *p < 0.05, **p < 0.01, and ***p < 0.001.

Among the functions of DENV NS3, ATPase activity is very important and is required for successful viral replication^34,35^. In this context, *in vitro* activity assays were performed to evaluate whether NS3 ATPase activity is affected by the presence of human GAPDH. A slight increase in ATPase activity was observed when full-length NS3 was incubated with 0.25, 0.5, 1 or 2 µM GAPDH. Only in the presence of 4 µM GAPDH was the ATPase activity significantly increased (Fig. 4A). Conversely, the ATPase activity of the helicase domain was significantly enhanced in the presence of GAPDH (Fig. 4B). The addition of 2 µM GAPDH to the enzymatic reaction resulted in an approximately 50% increase in activity. These results suggest that GAPDH can enhance the ATPase activity of the NS3 protein, especially the activity of the helicase domain.

### DENV2 NS3-GAPDH complex is structurally and energetically stable according to molecular docking

To understand at a structural level how the interaction of DENV2 NS3 with GAPDH can modulate the enzymatic activities of both enzymes, molecular docking simulations were used to build a three-dimensional model of the complex formed between full-length NS3 and its domains and GAPDH. Using the comparative modeling technique^36,37^, a 3D model of the full-length DENV2 NS3 protein was obtained based on the DENV4 NS3 crystal structure (PDB code 2VBC) at a 3.1-Å resolution^38^. These proteins share 77% amino acid identity, 89% amino acid similarity and 100% sequence coverage.

The final full-length DENV2 NS3 3D model had a DOPE score of −62416.70703 and an RMSD value of 0.3 Å between the model and full-length DENV4 NS3 (PDB code 2VBC). The Ramachandran plot showed that 89.7% of residues were in the most favored region, 9.1% were in additional allowed regions, 1.1% were in generously allowed regions and no residue was in disallowed regions.

**Figure 5:**
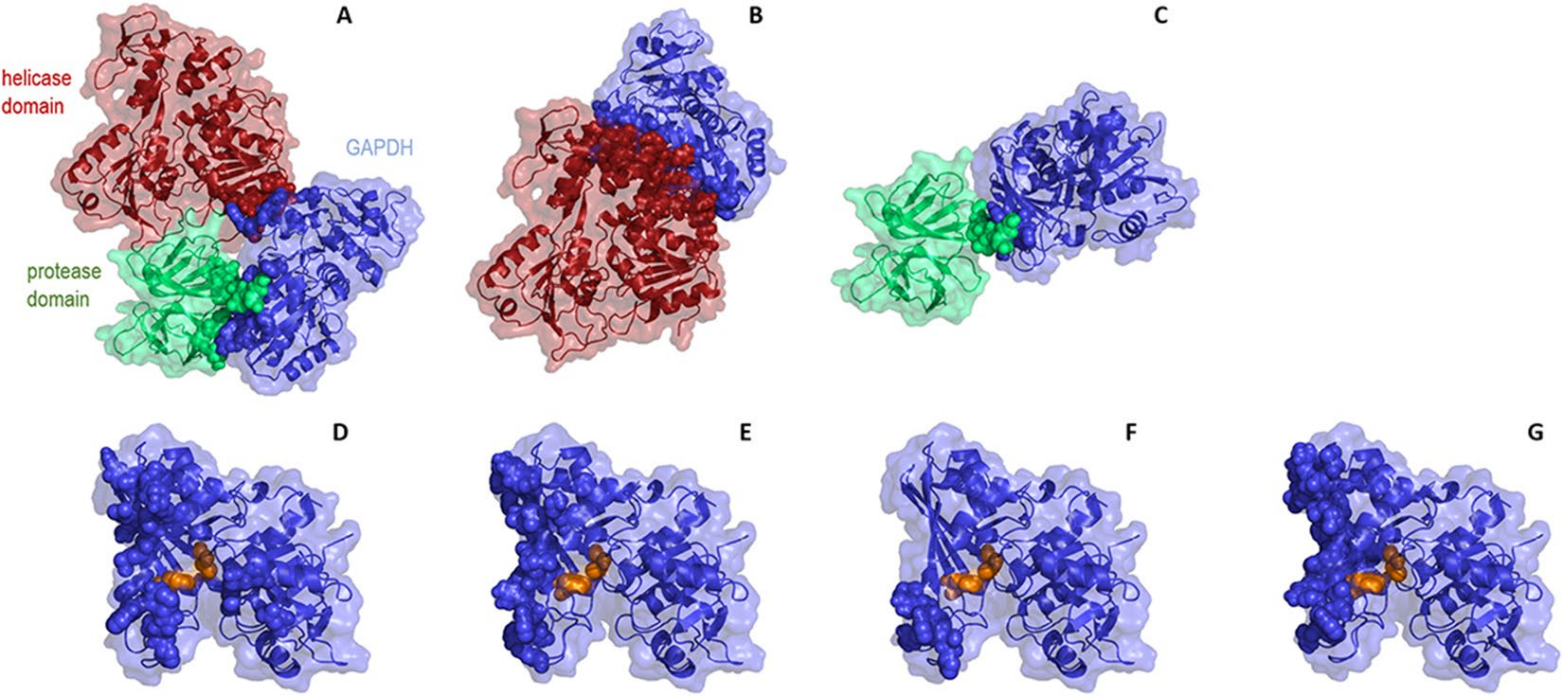
Representation of the DENV2 NS3-GAPDH complex by molecular docking. The 3D models of full-length DENV2 NS3 (green and red) and its separated protease (green) and helicase (red) domains were used as receptors. Human GAPDH monomer (blue) was used as the ligand. The amino acid residues found at protein interfaces are shown in spheres. The GAPDH catalytic residues, cysteine 152 (Cys152) and histidine 179 (His179), are shown in orange spheres. Human GAPDH binds full-length DENV2 NS3 (**A**) and its protease domain (**C**) using the same interaction interface. The binding site changes when GAPDH interacts with the NS3 helicase domain (**B**). Structures (**D**–**F**) correspond to (**A**–**C**) respectively, without the representation of the DENV2 NS3 structures to highlight the amino acid residues (blue spheres) of the GAPDH protein that are at the contact interface. The structure represented in G highlights the amino acid residues (blue spheres) of the GAPDH protein that are at the contact interface with DENV2 NS1, as described previously^39^ (the NS1 structure was not represented for better visualization). (**A**–**C**) are displayed in the same orientation among each other. (**D**–**G)** Are displayed in the same orientation among each other. Docking structures were generated by the web-based server Cluspro 2.0 and displayed with PyMOL.

The DENV2 NS3 3D model was used as the receptor for human liver GAPDH (PDB code 1ZNQ - O chain). Three different receptors were used for the docking simulations: full-length DENV2 NS3 (represented in green and red), its protease domain (green) and its helicase domain (red). The molecular docking results showed that GAPDH (blue) is predicted to bind the full-length NS3 protein and to its separate domains (Fig. 5 and Table 2). The interaction between GAPDH and the full-length NS3 protein was determined by 51 representatives structures with a center energy value of −881.6, suggesting that this interaction presents higher stability than when GAPDH binds the NS3 domains separately (Table 2). Center energy values of −807.0 and −802.0 were obtained for the GAPDH-NS3 protease and GAPDH-NS3 helicase interactions, respectively. These data corroborate the results obtained in the ELISA and SPR assays, which showed a higher affinity of GAPDH for full-length NS3 than for the individual NS3 domains.

**Table 2:** Score values for the full-length DENV2 NS3, the protease and helicase domains and human GAPDH docking.

System	Cluster number	Number of representatives	Balanced score	
full-length NS3 + GAPDH	1	51	Center	−881.6
			Lowest energy	−881.6
NS3 protease + GAPDH	0	86	Center	−807.0
			Lowest energy	−807.0
NS3 helicase + GAPDH	0	63	Center	−802.0

Interestingly, GAPDH binds the full-length NS3 protein and the protease domain using the same interaction interface (Fig. 5A,C). On the other hand, we observed that GAPDH binds the helicase domain using the other protein surface (Fig. 5B). The amino acids residues of GAPDH that are involved in the surface contact with the full-length DENV2 NS3 structure are: Asp81, Val101, Lys107, Val171, Glu172, Leu174, Thr176, Pro191, Ser192, Gly193, Lys194, Trp196, Lys215, Gly226, Lys227, Thr229, Gly230, Met231, Phe233, Thr246, Arg248, and Lys309 (Supplemental Data). However, the residues of GAPDH involved in the contact with the helicase domain are: Glu172, Leu174, Thr176, Asp189, Trp196, Arg197, Arg200, Asn205, Ile206, Pro208, Ser210, Lys215, Thr229, Meet231, Phe233, Asp244, Thr246, Arg248, Asn304, His306 and Phe307 (Supplemental Data). The residues of GAPDH involved in the contact with the protease domain are: Pro191, Ser192, Trp196, Arg197, Arg200, and Pro208 (Supplemental Data). Thus, the amino acids involved on the GAPDH surface depend on which parts of the DENV2 NS3 structure are involved in the interaction. In addition, we observed that the two catalytic residues of the GAPDH protein, cysteine 152 (Cys152) and histidine 179 (His179), are partially blocked when it interacts with the full-length NS3 but not with the helicase and protease domains (Fig. 5D–F), which might explain the stronger inhibitory effect of the full-length protein on GAPDH activity, as demonstrated in Fig. 3. The complete list of amino acid residues of the DENV2 NS3 and GAPDH proteins that are involved at the contact interface is listed in Supplemental Data.

### GAPDH interacts with the NS3 protein in DENV2-infected hepatic cells and NS3-transfected cells

**Figure 6:**
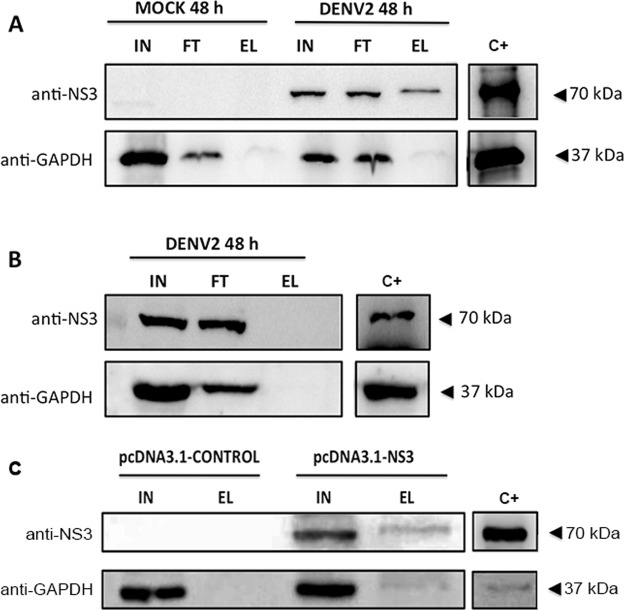
DENV2 NS3 interacts with the GAPDH protein in DENV2-infected Huh7.5.1 cells and in NS3-transfected BHK-21 cells. (**A**) Extracts from mock- or DENV2-infected Huh7.5.1 cells were coimmunoprecipitated with an anti-GAPDH rabbit polyclonal antibody covalently coupled to an amino-linked agarose resin. Elution was carried out using the elution buffer provided in the co-IP kit (Pierce). The input (IN), flow-through (FT) and eluted (EL) fractions were analyzed by western blotting using mouse anti-NS3 polyclonal and rabbit anti-GAPDH polyclonal antibodies. Gel bands of 37 and 70 kDa, which corresponded to the GAPDH and NS3 proteins, respectively, were observed in the elution fractions of the coimmunoprecipitation. (**B**) Extracts from DENV2-infected Huh7.5.1 cells were incubated with control IgG coupled to an amino-linked agarose resin. (**C**) Extracts from pcDNA3.1 or pcDNA3.1-NS3-transfected BHK-21 cells were coimmunoprecipitated with an anti-NS3 rabbit polyclonal antibody covalently coupled to an amino-linked agarose resin. The following steps were the same as described in (**A**). The purified human GAPDH protein (Abcam) and full-length DENV2 NS3 were used as positive controls (C+) as indicated in the Figure. The results presented are representative of three independent experiments. The blots were cropped from the same western blotting membrane. Full-length blots/gels are presented in Supplementary Data.

To assess whether the interaction between the NS3 and GAPDH proteins occurs in infected human cells, Huh7.5.1 cells were first infected with DENV2 strain 16681 at an multiplicity of infection (MOI) of 2 for 48 h. Protein extracts from mock- or DENV2-infected Huh7.5.1 cells were then loaded onto a column in which an anti-GAPDH polyclonal antibody had been previously immobilized to the resin. After the incubation period, the resin was washed 4 times with immunopreciptation (IP) lysis buffer (Pierce, USA) and the washed fractions were applied to an SDS-polyacrylamide gel. We observed that the number of washes was sufficient to remove non-specific proteins (data not shown). Elution fractions were then analyzed by western blotting using anti-NS3 mouse polyclonal and anti-GAPDH rabbit polyclonal antibodies. Two bands of approximately 37 and 70 kDa, which corresponded to the GAPDH and NS3 proteins, respectively, were obtained, indicating that the proteins coimmunoprecipitated (Fig. 6A). The weak band of approximately 37 kDa observed in both mock- and DENV2-infected cell extracts is likely because of the strong interaction between the anti-GAPDH polyclonal antibody (used in the preparation of the Co-IP column) and GAPDH protein, which is not disrupted with the co-IP elution buffer (Fig. 6A). A co-IP assay using a non-specific IgG_1_ coated in resin did not yield any specific bands for the human GAPDH and NS3 proteins (Fig. 6B). To rule out the possibility that other DENV proteins than NS3 would also be involved in the interaction with GAPDH protein (i.e., NS1 protein^39^), and thus interfering in the co-IP results, the protein extract of NS3-transfected BHK-21 cells was also subjected to the co-IP assay (Fig. 6C). Different from the co-IP assay of the DENV-infected cells (Fig. 6A), here the protein extract was loaded onto a column in which an anti-NS3 polyclonal antibody (prepared in-house) had been previously immobilized to the resin. As expected, a 37-KDa band corresponding to the GAPDH protein in the elute fraction of NS3-transfected cell extract was observed in the western blotting gel detected by anti-GAPDH polyclonal antibody. The same band was not detected in the control cell extract, confirming that GAPDH specifically interacts with DENV NS3 protein in the absence of other viral proteins. Altogether, these findings obtained by the co-IP assays clearly indicate that the interaction between human GAPDH and DENV2 NS3 can occur during DENV infection, confirming the result obtained by the yeast two-hybrid system.

### Intracellular localization of GAPDH changes during DENV2 infection

**Figure 7:**
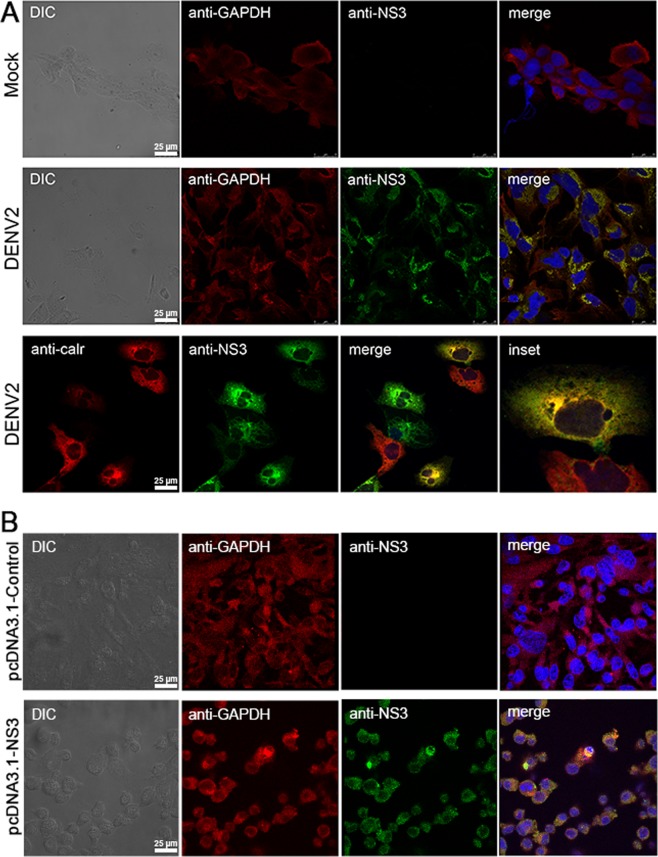
Human GAPDH relocalizes to ER membranes and colocalizes with NS3 in DENV2-infected Huh7.5.1 cells and in NS3-transfected BHK-21 cells. (**A**) Huh7.5.1 cells were mock- or DENV2-infected at an MOI of 2, fixed 48 h post-infection and subjected to double-immunofluorescence staining with polyclonal anti-GAPDH (red stained), polyclonal anti-NS3 (green stained) or monoclonal anti-calreticulin (red stained) antibodies. GAPDH shows diffuse localization in mock-infected cells. The distribution of GAPDH in DENV2-infected cells shows intense perinuclear signals that coincide with NS3 localization, as revealed by the merged images. The colocalization of NS3 with calreticulin, an ER marker, was observed in DENV2-infected cells. Nuclei were counterstained with DAPI (blue). (**B**) BHK-21 cells were transfected with pcDNA3.1 (negative control) or pcDNA3.1-NS3, fixed 48 h post-transfection and subjected to double-immunofluorescence staining with polyclonal anti-GAPDH (red stained), polyclonal anti-NS3 (green stained). Nuclei were counterstained with DAPI (blue).

NS3 is present in the cytoplasm, specifically in virus-induced endoplasmic reticulum (ER) vesicles, where DENV replicates. To evaluate whether GAPDH, a canonical cytosolic protein, colocalizes with NS3 and whether its localization changes during DENV infection, Huh7.5.1 cells were mock- or DENV2-infected with an MOI of 2, immunostained and analyzed by confocal microscopy. The results showed that the distribution of GAPDH was predominantly cytoplasmic in mock-infected cells (Fig. 7A). Conversely, strong GAPDH staining was observed in perinuclear regions coincident with the position of the ER network. The merged images revealed that the GAPDH and NS3 proteins colocalized in these regions in DENV2-infected cells at 48 h post-infection (Fig. 7A), suggesting that GAPDH localization might be modulated upon DENV2 infection. The intracellular localization of GAPDH protein was also analyzed in NS3-transfected cells (Fig. 7B). Similarly to the result from the GAPDH distribution obtained in DENV-infected cells, GAPDH protein also stains more strongly in perinuclear regions where NS3 protein is normally found (Fig. 7B, merge panel).

### DENV3 NS3 and human GAPDH costain in hepatic tissue

**Figure 8:**
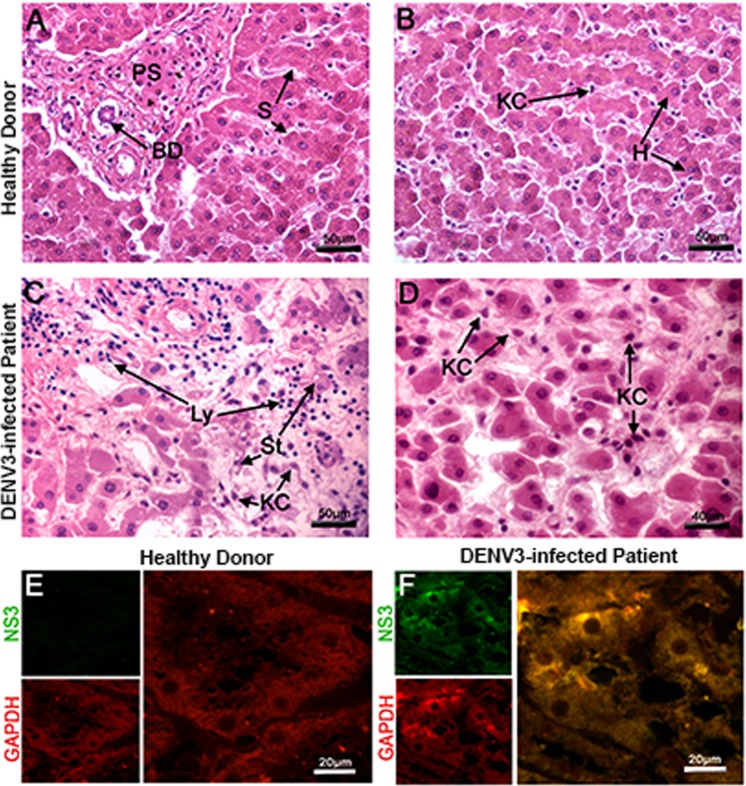
DENV3 generates hepatic steatosis, and the DENV3 NS3 protein colocalizes with GAPDH in hepatic tissue from a fatal dengue case. Liver sections from a healthy donor (**A**–**B**) or a DENV3-infected patient (**C**–**D**) were stained with hematoxylin and eosin for histological analysis or incubated with purified mouse polyclonal anti-NS3 helicase (green fluorescent) and rabbit polyclonal anti-GAPDH antibodies (red fluorescent) (**E**–**F**). Hematoxylin-and-eosin-stained liver sections from the non-dengue case show a normal appearance (**A**–**B**), whereas liver sections from the infected fatal case presented hepatic injuries in the form of microsteatosis (St) (**C**) and Kupffer cell and lymphocyte infiltrates (**D**). The NS3 protein was not detected in control tissue (**E**), but GAPDH and DENV3 NS3 colocalized in infected hepatic tissue (**F**), as observed in the merged images of hepatocytes and Kupffer cells. Hepatocytes (**H**); Sinusoidal Capillary (**S**); Biliary Duct (BD), Portal Space (PS); Steatosis (St); Kupffer cells (KC); and Lymphocytes (Ly).

To evaluate whether GAPDH and DENV NS3 also colocalize in infected liver tissue, immunofluorescence assays and histopathological analyses were carried out using hepatic tissues obtained from a fatal dengue case of DENV3-infected patient or from a healthy donor. The hepatic tissue presented a liver parenchyma lesion based on the presence of lipid accumulation within hepatocytes, which showed small fat vacuoles around the nucleus (microsteatosis) in Kupffer cells, and lymphocyte infiltrates were visible around the portal space and sinusoidal capillary (Fig. 8C,D). As expected, these lesions were not found in hepatic tissues from the healthy donor (Fig. 8A,B).

GAPDH was detected by immunofluorescence (red fluorescent) in the cytoplasm and nucleus of the hepatocytes and Kupffer cells in the liver samples from the fatal dengue case and the control (Fig. 8E,F). DENV3 NS3 proteins were identified (green fluorescent) mainly in the cytoplasm of the hepatocytes and Kupffer cells, only in the liver samples from the fatal dengue case (Fig. 8F). GAPDH and DENV2 NS3 were colocalized in hepatic tissue from the fatal dengue case, as observed in the merged image of numerous Kupffer cells and hepatocytes (Fig. 8E). Taken together, these results suggest that the human GAPDH-DENV2 NS3 interaction may be involved in hepatic metabolic alterations, which may contribute to the appearance of steatosis in dengue-infected patients.

### DENV3 NS3 reduces intracellular GAPDH glycolytic activity

**Figure 9:**
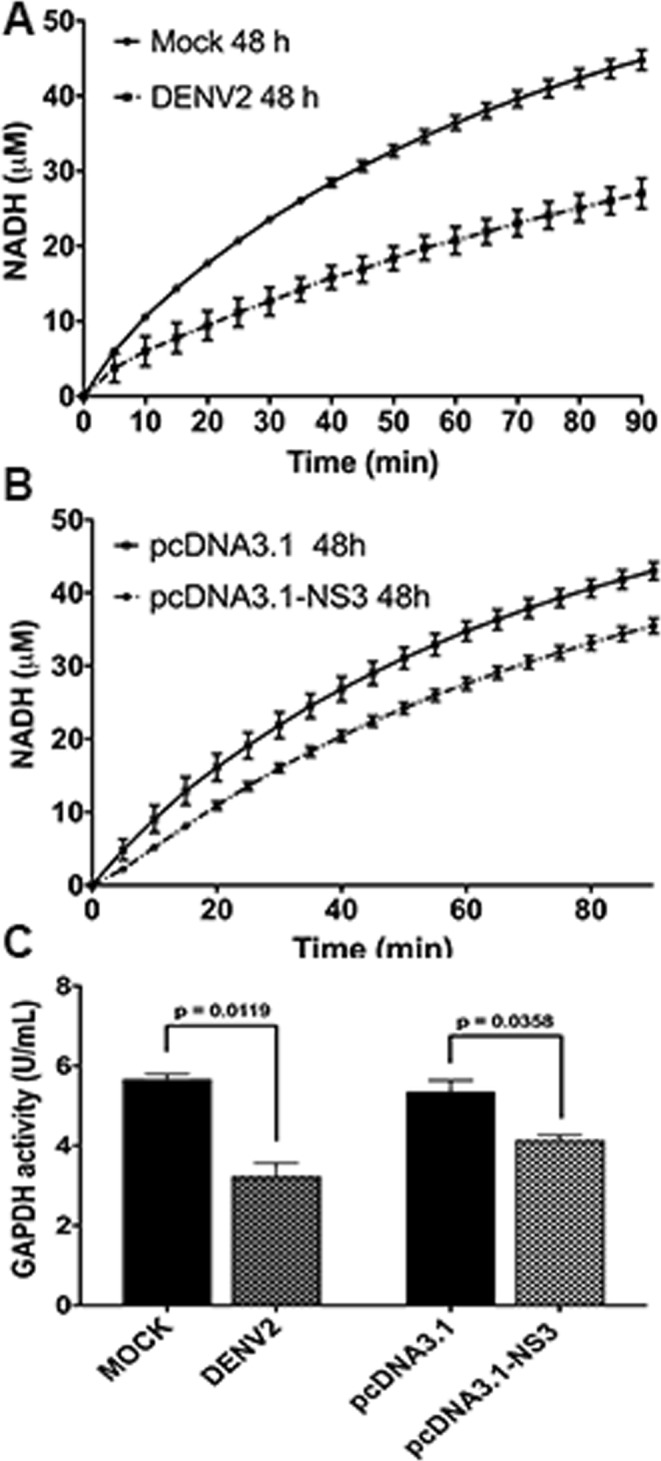
DENV2 NS3 protein decreases the intracellular GAPDH glycolytic activity in DENV2-infected and NS3-transfected cells. Intracellular GAPDH activity was monitored in a coupled reaction with TPI, in which dihydroxyacetone phosphate was converted into glyceraldehyde-3-phosphate, the GAPDH substrate. Kinetics of NADH were monitored by the increase in absorbance at 340 nm every 1 min for 90 min, using 50 µg of the cell extract of DENV2-infected (**A**) or NS3-transfected BHK-21 cells (**B**) as the source of the enzyme. The conversion of absorbance units into NADH production was determined by molar absorptivity of NADH (6.22 mM^−1^ cm^−1^). The concentration of NADH was converted into GAPDH activity, considering that one unit of enzyme activity corresponds to the reduction of 1 µM of β-NAD/min (**C**).

Based on the results presented above, it was important to investigate whether GAPDH glycolytic activity is decreased in DENV2-infected and NS3-transfected cells. The cytosolic fractions of the cellular extract from mock- or DENV2-infected BHK-21 cells were used to analyze intracellular GAPDH activity measured in a coupled reaction with triose phosphate isomerase (TPI) enzyme. After 48 h of infection, the NADH production was significantly increased in DENV2-infected cells compared with that in the mock-infected cells (Fig. 9A). The level of NADH production was then converted into GAPDH activity, revealing a decrease of about 50% in GAPDH activity after 48 h of DENV2 infection (Fig. 9C; *P* < 0.05).

The direct influence of NS3 protein on GAPDH activity was also evaluated by measuring the GAPDH glycolytic activity in BHK-21 cells transfected with either the empty pcDNA3.1 plasmid or the pcDNA3.1-NS3 plasmid. An increase in the level of NADH production was measured in the NS1-expressing cells at 48 h (Fig. 9B) after transfection. GAPDH activity was decreased about 25% in the NS3-expressing cells (Fig. 9C; *P* < 0.05). These results indicate that both DENV2 and NS3 expression decrease GAPDH activity at 48 h post-infection or post-transfection.

## Discussion

In our study, we identified 47 different cellular proteins from a liver library that potentially interact with DENV2 NS3 *in vivo*. The majority of these proteins are involved in essential metabolic processes, such as purine biosynthesis, transcriptional regulation, the inhibition of serine protease activity, the regulation of lipid metabolism, oxidative phosphorylation, and glycolysis. In a previous work published by Khadka and colleagues, 139 interactions between human proteins and eight of the ten DENV proteins (M, NS1, NS2A, NS2B, NS3, NS4A, NS4B and NS5) were identified, including their fragments, by using a yeast two-hybrid system and a liver cDNA library^40^. Among these interactions, only the fibrinogen beta chain (FGB) protein was also detected in the yeast two-hybrid screening reported here. FGB is an important factor involved in the coagulation cascade and is centrally located in the human protein interaction network by binding DENV NS3, DENV NS1 and other host proteins^40^.

Moreover, we identified two proteins belonging to the serpin superfamily, SERPINC1 and SERPINA3. These proteins are plasma serine proteases that inhibit thrombin and other activated serine proteases of the coagulation system, regulating the blood coagulation cascade. Interestingly, Khadka’s group detected another protein of this same family, SERPIND1, which is also located at the center of the protein interaction network and which binds several human proteins and DENV proteins, such as NS1, NS3 and NS2B^40^.

In this report, we highlighted the interaction of DENV NS3 with GAPDH, a key protein of the glycolytic pathway. To date, several studies have demonstrated that GAPDH is a multifunctional protein. Despite its classic function in energy production, GAPDH is involved in unrelated cellular pathways, such as oxidative stress^41^, apoptosis induction and regulation^42,43^, and membrane trafficking and fusion^44^.

We demonstrated through co-IP experiments followed by western blotting that GAPDH interacts with intracellular NS3, as identified by a two-hybrid system. The formation of a complex by DENV NS3 and GAPDH was also confirmed by *in vitro* assays, such as ELISA and SPR, using purified proteins. GAPDH directly bound full-length NS3, as well as the NS3 helicase and protease domains; however, the affinities of these interactions differed significantly. Full-length NS3 bound GAPDH more strongly than the separate NS3 domains, with an affinity in the low micromolar range (*K*_*D*_ = 0.31 µM). This result indicates a mildly strong interaction, which is characteristic of multifunctional proteins that dynamically bind many partners involved in several cellular processes^45^.

In addition to its established cytosolic localization, GAPDH is also found in the nucleus, which is dependent on the acetylation of lysine residues, in mitochondria or associated with the plasma membrane, ER or Golgi apparatus^46,47^. Moreover, GAPDH interacts with viral RNAs and possesses RNA helix destabilizing activity^48^, probably by preferentially binding single-stranded RNA regions^49,50^. Here, we demonstrated that DENV2 infection redistributes GAPDH from the cytoplasm to the perinuclear region in Huh7.5.1 cells, where the protein localizes with NS3. This redistribution was also observed in the NS3-transfected BHK-21 cells. Such observation is supported by the fact that DENV2 infection is responsible for GAPDH redistribution in infected BHK-21 cells^39^. Our results also suggest that GAPDH is shifted to the replicative complex in the ER during DENV2 infection and that the activity of GAPDH might be decreased by its interaction with the NS3 protein. In addition, Japanese encephalitis virus (JEV) infection changes the cellular localization of GAPDH from the nucleus to the cytosol, where it directly binds the 3′ termini of plus- and minus-strand RNAs and colocalizes with the NS5 protein in JEV-infected cells, suggesting that GAPDH participates in the JEV replicative cycle^51^.

Previous work from our group showed that DENV infection modulates GAPDH glycolytic activity during the early steps of infection. DENV2 NS1 binds GAPDH, and this interaction increases GAPDH activity both *in vitro* and *in vivo*^39^. Additionally, another proteomic study performed on hepatocytes transfected with the DENV2 NS1 protein showed equal levels of GAPDH in control and NS1-expressing cells, suggesting that both NS1 and NS3 interact with GAPDH and modulate its activity but do not affect its protein expression level^52^. In addition, a recent work demonstrated that the (siRNA)-mediated knockdown of human GAPDH in Huh-7.5.1 cells inhibits intracellular DENV2 E glycoprotein production in infected cells^53^.

Here, we demonstrated that full-length NS3 reduces GAPDH activity *in vitro*. Although this result appears to contradict previous reports on the effect of the NS1 protein on GAPDH activity at first glance^39^, it can be explained in terms of both thermodynamic and kinetic processes. The former process is based on the fact that the K_D_ of 0.31 µM calculated for the GAPDH-NS3 complex is almost 10-fold smaller than the K_D_ estimated of about 2–3 µM for GAPDH-NS1 complex [according with the ELISA result^39^ and SPR assays (unpublished data)]. Moreover, both the NS1 and NS3 proteins bind the same surface of the GAPDH protein, as demonstrated by molecular docking (Fig. 5G and Supplemental Data), suggesting that these proteins compete with each other for the same binding surface. As NS3 protein has almost 10-fold higher binding affinity to GAPDH than NS1 protein, it is expected that the GAPDH-NS3 complex prevail. Nonetheless, NS3 is a crucial player in viral replication, especially in the beginning of this process, forming complexes with several other macromolecules such as NS2B, NS5, and RNA, for example, which can compete with GAPDH for the same binding site. In other words, very likely, there are not enough NS3 molecules free to interact with GAPDH. Here, the kinetic process plays a very important role. Based on our previous results, GAPDH activity increases in the first 24 h DENV2 post-infection but decreases at 48 h^39^, which clearly suggests that this enzymatic activity is time-dependent. Hence, we hypothesize that the regulation of GAPDH activity by the NS1 protein predominates in the first 24 h, while NS3 is mainly involved in viral replication, increasing the GAPDH activity. However, as the time of infection proceeds, this regulation turns to be controlled by the NS3 protein, decreasing the GAPDH activity. This phenomenon might be caused by the NS3/NS1 concentration ratio, which changes over time, as the NS1 protein is secreted, while the NS3 concentration remains nearly constant. To corroborate this hypothesis, we repeated the same experiment that was previously reported by our group^39^ to detect the NS3 protein in this study (Supplemental Data). Indeed, the concentration the NS3 protein did not change significantly over time during infection, but the NS1 secretion is increased, avoiding its intracellular accumulation.

GAPDH enzyme participates in the sixth step of glycolysis; thus, the reduction in its activity causes the accumulation of metabolic intermediates, which probably leads to alternative routes of the glycolysis pathway. One hypothesis is that the decreased GAPDH activity can increase the concentration of glucose-6-phosphate, which is converted into ribose via the pentose phosphate pathway, thereby enhancing nucleotide availability for DENV RNA synthesis.

Another hypothesis involves the conversion of glyceraldehyde-3-phosphate into its isomer, dihydroxyacetone phosphate, which is reduced to glycerol-3-phosphate. Phosphatidic acid production can provide more phospholipids for the construction of new inner membranes, such as the ER and Golgi apparatus, which are required for viral replication and assembly. Moreover, phosphatidic acid is a precursor for diverse metabolic reactions, one of which is its conversion to diacylglycerol, which is responsible for cellular fat storage, thereby promoting the steatosis events frequently observed in DENV-infected hepatocytes. This hypothesis is in accordance with a previously published work^54^, in which DENV infections altered lipid homeostasis. In addition, our finding that GAPDH and DENV3 NS3 colocalize extensively in regions containing hepatocytes and Kupffer cells in the liver tissue of a fatal dengue case supports our hypothesis regarding the involvement of NS3 and GAPDH in viral replication. Further, this co-localization proved that this interaction does not occur only *in vitro*. In other words, the ratio of NS3/NS1 concentrations over time modulates GAPDH activity such that during the first 24 h of post-infection, the glycolytic pathway is activated to produce more energy for viral replication, and after this period of time, GAPDH activity is down-regulated, and cellular metabolism shifts to the synthesis of several metabolites that are also important for viral infection and propagation.

Moreover, GAPDH binding was able to increase the ATPase activity of full-length NS3 and the NS3 helicase domain, consequently inducing increased rates of helicase activity. ATP hydrolysis is a key step of the viral replicative cycle, providing the energy source for the dissociation of double-stranded RNA replication intermediates and other viral processes. Thus, we suggest that the direct binding between GAPDH and NS3 could be responsible for increasing the amount of single-stranded RNA to be used as templates for new viral RNA synthesis, which may result in higher rates of replication and virion assembly.

In summary, our results demonstrate that DENV NS3 directly interacts with human GAPDH, a multifunctional protein that possesses several functions in addition to its glycolytic role. The DENV2 NS3-GAPDH interaction results in increased NS3 ATPase activity and decreased GAPDH glycolytic activity. Thus, we can assume that such an interaction contributes to the unwinding of double-stranded RNA and to the virus-induced vesicle proliferation that is needed for RNA replication and virion assembly. This hypothesis is corroborated by previous studies that demonstrated that the interaction between DENV NS3 and the nuclear receptor binding protein (NRBP) may be involved in membrane structure formation during infection^55^. On the other hand, decreased GAPDH glycolytic activity may be an important mechanism by which DENV alters host lipid homeostasis. The redistribution of GAPDH in DENV2-infected hepatocytes is likely to be associated with its participation in the replication complex along with NS3 protein. Our study offers a new perspective on the role of metabolic enzymes in DENV infection.

## Materials and Methods

### Cloning the ns3 gene into the pGBKT7 and pcDNA3.1-mycHisA plasmids

For the pGBKT7 cloning, the full-length *ns3* gene was amplified by polymerase chain reaction (PCR) from cDNA from the DENV2 New Guinea C (NGC) strain using the forward primer 5′-AATAGGATCCATGCTGGAGTATTGTGGGATGTCC-3′, which contains a *Bam*H I restriction site (underlined), and the reverse primer 5′-TATTGCGGCCGC*TTA*CTTTCTTCCAGCTGCAAACTCC-3′, which contains a *Not* I restriction site (underlined) and a TAA stop codon (italics). The PCR conditions were as follows: 94 °C for 2 min, followed by 35 cycles of 94 °C for 30 s, 58 °C for 1 min and 68 °C for 2 min, and a final extension of 68 °C for 7 min. The amplified gene was cloned in frame with the GAL4 DNA binding domain (BD) of the yeast expression vector pGBKT7 (Clontech, USA) to construct the bait plasmid (pGBKT7-NS3). For the cell transfection experiments, the forward primer 5′-AATAGGTACC**ACCATGG** CTGGAGTATTGTGGGATGTCC-3′, which contains a *Kpn I* restriction site (underlined) and a Kozak sequence (bold), and the reverse primer with the same DNA sequence as mentioned above were used for PCR amplification of NS3 gene using the pGBKT7-NS3 plasmid obtained above as DNA template. The PCR conditions were the same as described above. After the digestion of both the PCR product and the plasmid pcDNA3.1-mycHisA (Invitrogen, USA) with the restriction enzymes *Kpn I* and *Not I* (NEB, USA), followed by the DNA purification and ligation steps, the full-length NS3-carrying construct (pcDNA3.1-NS3) was obtained.

### Yeast two-hybrid screening

Using the full-length NS3 protein as bait, a yeast two-hybrid screen was performed against a human liver cDNA library fused to the GAL4 activation domain (AD) using the pACT2 vector and the Matchmaker GAL4 Two-Hybrid System 3 (Clontech, USA). The AH109 yeast strain was transformed with the pGBKT7-NS3 plasmid using the lithium acetate method and then grown in synthetic defined (SD) medium lacking tryptophan (SD–Trp). Autoactivation of the *HIS3* reporter gene was confirmed by the growth of clones in SD lacking histidine, leucine and tryptophan (SD–His–Leu–Trp). The transformed cultures were then plated onto SD–His–Leu–Trp and SD–Ade–His–Leu–Trp media to select putative positive clones. The expression of the *lacZ* reporter gene was evaluated using a β-galactosidase assay (colony-lift filter) using the X-gal substrate on nitrocellulose membranes. To isolate the DNA plasmid from each positive yeast colony, the Zymoprep Yeast Plasmid Miniprep Kit (Zymo Research, USA) was used. Then, to isolate only the pACT2-cDNA library (prey), the DNA plasmid from yeast was transferred to *E. coli* by electroporation. To eliminate false-positive clones, a plasmid-linkage assay was also performed, in which AH109 yeast cells that were cotransformed with the pGBKT7-NS3 plasmid and each pACT2-cDNA library were visualized again on SD–His–Leu–Trp and SD–Ade–His–Leu–Trp media and evaluated using the β-galactosidase assay. The positive plasmids were sequenced and their gene sequences were analyzed using the BLASTN and BLASTX software available at NCBI (www.ncbi.nlm.nih.gov).

### GAPDH and NS3 proteins and antibodies

Human GAPDH was purchased from Abcam (USA). The full-length *ns3* gene, the *ns3* helicase gene (from amino acid 169 to 619) and the *ns3* protease gene (from amino acid 1 to 185) from the cDNA of the DENV2 NGC strain were successfully cloned into the *E. coli* expression vector pET21dEZ^56^. To express the recombinant full-length NS3 protein and its protease domain, competent Rosetta [λDE3] *E. coli* cells were transformed with the recombinant plasmids by heat shock. The helicase domain was expressed in BL21 [λDE3] *E. coli* cells. The expression and purification steps for the full-length NS3 and the protease and helicase domains were performed as described previously^56^. A polyclonal antiserum against the purified full-length NS3 protein or its helicase domain was raised in mice^57^ and in rabbit, and purified using HiTrap NHS activated HP columns (GE Healthcare, USA), to which the purified NS3 protein was covalently coupled.

### Enzyme-linked immunosorbent assay (ELISA)

The wells of a 96-well microtiter plate (Nalge Nunc, Denmark) were coated overnight at 4 °C with a 50 µg/mL concentration of each purified NS3 protein (5 µg of full-length, protease or helicase domains) in PBS buffer (8.06 mM sodium phosphate, 1.94 mM potassium phosphate, 2.7 mM KCl, and 137 mM NaCl) at pH 7.4. The plates were blocked for 1 h at room temperature with 1% bovine serum albumin (BSA) in PBST (0.05% Tween 20 in PBS) and then washed three times with PBST. This step was performed after each incubation period. The wells were then incubated for 3 h with serial dilutions of GAPDH (Abcam, USA) or BSA (as a negative control), with concentrations ranging from 1.6 µg/mL to 100 µg/mL, at room temperature. Subsequently, the wells were incubated for 2 h at room temperature with an anti-GAPDH polyclonal antibody (Abcam, USA) diluted in PBS and then incubated with a horseradish peroxidase-conjugated anti-IgG rabbit antibody (Promega, USA). After 20 min at room temperature, the reactions were visualized with OPD (Sigma Aldrich, USA) and H_2_O_2_ as the substrates and 12.5% H_2_SO_4_ as the quencher. The reactions were monitored by measuring the absorbance at 490 nm. The optical density (OD) measurements were normalized using the mean value of the blank control (no interacting partner, PBS only). A two-way ANOVA followed by Bonferroni post-test was used to calculate statistical significance between the control and NS3-containing curves. The bar graphs represent the area under the curve (AUC) for each NS3 construct compared to its corresponding negative control (BSA). The error bars indicate the standard deviation from three independent experiments, and the *p* values show significant differences between NS3 constructs and BSA curves, calculated by unpaired Student’s *t* test.

### SPR measurements

Surface plasmon resonance (SPR) measurements were performed using a Biacore X instrument (GE Healthcare, USA). GAPDH was covalently immobilized onto the dextran matrix of a CM5 sensor chip via the primary amine groups (amine coupling kit; GE Healthcare, USA). The carboxymethylated dextran surface was activated by the injection of a mixture of 0.2 M N-ethyl-N′-(diethylamino-propyl)carbodiimide and 0.05 M N-hydroxysuccinimide. The ligand was injected in 10 mM sodium acetate buffer (pH 5.5). The remaining N-hydroxysuccinimide esters were blocked by injecting 1 M ethanolamine hydrochloride (pH 8.5). All immobilization steps were performed at a flow rate of 10 μL/min in 10 mM HEPES, 150 mM NaCl, 3 mM EDTA, and 0.005% P20 (GE Healthcare, USA) (pH 7.4). The immobilization level for GAPDH was 9,150 RU. No protein was immobilized on the control flow cell that underwent the activation and blocking steps. Binding experiments were performed at 25 °C at a flow rate of 15 μL/min in 10 mM HEPES and 150 mM NaCl (pH 7.4). The full-length NS3 protein and the NS3 protease and NS3 helicase domains were injected at different concentrations, ranging from 0.06 µM to 4.0 µM. The data were double-referenced by subtracting the control flow cell signal of a blank run (buffer only). In all experiments, association phases were carried out for 120 s and dissociation phases were carried out for 280 s. The surface was regenerated with pulses of 0.01 M glycine (pH 2.0). The data were analyzed by global fitting to a 1:1 Langmuir binding model of both the association and dissociation phases for several concentrations simultaneously using the BIAevaluation 4.1 software (BIAcore, USA). In each case, the presented data were obtained with a statistical χ^2^ value < 2. The apparent equilibrium dissociation constants (K_D_) were calculated from the ratio of the dissociation and association rate constants (k_d_/k_a_).

### Measurements of enzymatic activities

GAPDH enzymatic activity was measured in a 96-well microtiter plate using the following medium: 50 mM Tris-HCl, pH 7.4, 2 mM MgCl_2_, 1 mM ATP, 1 mM EDTA, 0.15 mM β-NADH, 13 units/mL phosphoglycerate kinase (PGK), and 1 µM recombinant human GAPDH protein (Abcam, USA). The wells were then incubated with different concentrations of full-length NS3, the NS3 protease domain or the NS3 helicase domain and BSA (negative control). The reactions were started by the addition of 5 mM 3-phosphoglycerate. For all reactions, the baseline was monitored for 3 min. The fluorescence intensity was measured on a *SpectraMax M5*^*e*^ microplate reader (Molecular Devices, USA) at Ex/Em = 352/464 nm.

### ATP hydrolysis assays

ATPase activity in the presence of GAPDH was determined using a colorimetric assay that measures the hydrolysis of ATP to ADP + Pi using a standard curve with known Pi concentrations^58^. Briefly, 40 µL of buffer A (40 mM Tris-HCl pH 7.5, 5 mM DTT, 5 mM MgCl_2_, and 100 mM KCl) containing 0.3 µM of purified NS3 helicase domain or 0.6 µM of full-length NS3 protein was added to the wells of a 96-well microtiter plate containing serial dilutions of the human GAPDH protein or BSA (from 4 μM to 0.25 μM). After 15 min, the reactions were started with 1 mM ATP diluted in buffer A, followed by incubation at 30 °C for 60 min. Then, the generated free Pi was quantified after the addition of 80 µL of an ammonium molybdate solution and 40 µL of reducing agent. These two compounds react with free Pi to generate a blue-colored product that was read at 660 nm on a *SpectraMax M5*^*e*^ microplate reader (Molecular Devices, USA).

### Molecular modeling and docking

The predicted 3D model of the full-length DENV2 NS3 protein was obtained by comparative modeling. Analyses of secondary structures were carried out using the PSIPRED program^59^. Sequence alignment and model building were carried out using the ‘align2d’ module of MODELLER^60,61^, a global dynamic programming algorithm that takes into account structural information from the template when constructing the alignment.

Several candidate 3D models of the DENV2 NS3 protein were constructed based on the crystal structure of full-length DENV4 NS3 (PDB code 2VBC)^35^, all of which had a GA341 score of 1.0, which means that the obtained models are comparable to low-resolution X-ray structures^62^. The models were ranked using discrete optimized protein energy (DOPE) scores by MODELLER, and checked and validated using Ramachandran plots by PROCHECK program^63^. The root mean square deviation (RMSD) between the models and the template, as well as other analyses, including the 3D model visualization, was determined in PyMOL^64^.

Molecular docking assays were carried out with the ClusPro 2.0 webserver^65–68^. The 3D model of DENV2 NS3 was used as a receptor for human GAPDH (PDB code 1ZNQ - O chain). The analysis of the amino acids located at the protein interface was obtained by InterProSurf Protein-Protein Interaction Server^69^. The Supporting Information was rendered in PyMOL^64^.

### Cell culture, DENV2 infection and transient-transfection assay

Huh7.5.1 cells, a subline derived from the hepatocyte Huh7.5 cell line, were cultured in high-glucose DMEM (Dulbecco’s Minimal Essential Medium) supplemented with 10% (v/v) fetal bovine serum (Invitrogen, USA), 0.22% sodium bicarbonate and 0.2% HEPES, pH 7.4, 0.1 mM nonessential amino acids, 2 mM L-glutamine (Gibco, USA) and penicillin-streptomycin (Invitrogen, USA) in a humid 5% CO_2_ incubation chamber at 37 °C. After 2 days, the Huh7.5.1 cells were mock-infected or infected with DENV2 strain 16681 at a MOI of 2. All experiments were performed at 48 h post-infection. DENV2 strain 16681 was propagated in C6/36 cells for 7–9 days. The culture media were recovered and stored at −80 °C. For the transient-transfection assays, BHK-21 cells were seeded in a 6-well plate at a density of 2 × 10^5^ cells/well and cultured in α-MEM with 10% FBS for 1 day before transient transfection. A mixture containing 2 µg of the empty pcDNA3.1-mycHisA plasmid (negative control) or the pcDNA3.1-NS3 plasmid (carrying the full-length *ns3* gene), Lipofectamine 2000 (Invitrogen), and Opti-MEM medium (Gibco) was added to the cell culture. After 5 h of incubation, the transfection medium was replaced by fresh α-MEM with 10% FBS, and the culture was maintained for 48 h in a humid chamber at 37 °C with 5% CO_2_.

### Coimmunoprecipitation and western blotting

At 48 h post-infection, mock-infected and DENV2-infected Huh7.5.1 cells (10^6^) were washed twice with PBS buffer prior to detachment using trypsin. The suspensions were centrifuged at 1,200 × g for 10 min, and the cell pellets were resuspended in IP lysis buffer (Pierce, USA) containing 25 mM Tris-HCl (pH 7.4), 150 mM NaCl, 1 mM EDTA, 1% NP-40, 5% glycerol, protease inhibitors (1 mM phenylmethylsulfonyl fluoride, 0.02 mM pepstatin A, 0.01 mM leupeptin, 0.01 mM aprotinin, 0.01 bestatin, and 0.02 mM E64), 0.025 mg/mL RNase and 0.025 mg/mL DNase. The cell extracts were incubated on ice for 15 min and centrifuged at 13,000 × g for 10 min at 4 °C. The supernatants were collected and subjected to co-IP assays, which were performed using the Pierce Co-IP Kit (Pierce, USA). Approximately, 30 µg/column of an anti-GAPDH rabbit polyclonal antibody (Abcam, USA) or an IgG1 isotype control antibody was attached to the AminoLink Plus coupling resin, followed by equilibration and incubation with 1 mg of mock-infected or DENV2-infected Huh7.5.1 cell extracts for 24 h at 4 °C. Each column was washed with IP lysis buffer and the protein complexes bound to the antibodies were eluted with elution buffer (glycine, pH 2.8).

For the coIP assays of NS3-transfected cells, BHK-21 cells were transfected using Lipofectamine 2000 reagent (Thermo Fisher, USA). At 48 h post-transfection, approximately 10^6^ pcDNA3.1- (negative control) and pcDNA3.1-NS3-transfected BHK-21 cells were washed twice with PBS buffer prior to detachment using trypsin. The protein extract was prepared following the same protocol used for preparation of protein extract of the DENV2-infected cells. Approximately, 50 µg/column of an anti-NS3 rabbit polyclonal antibody (Abcam, USA) was attached to the AminoLink Plus coupling resin, followed by equilibration and incubation with 1 mg of pcDNA3.1- or pcDNA3.1-NS3-transfected BHK-21 cell extracts overnight at 4 °C. Each column was washed with IP lysis buffer, and the protein complexes bound to the antibodies were eluted with elution buffer (glycine, pH 2.8).

The elution fractions obtained from three independent co-IP assays were pooled and the proteins were precipitated with 100% trichloroacetic acid to a final concentration of 10%. The pelleted proteins were resuspended in 30 µL of SDS-PAGE loading buffer, and half of this volume was loaded onto a 10% SDS-polyacrylamide gel and transferred onto a Hybond ECL nitrocellulose membrane (GE Healthcare, USA). The membrane was then blocked with 5% nonfat milk diluted in TBST (0.1% Tween 20 in TBS [25 mM Tris-HCl, pH 7.6, 3 mM KCl, and 140 mM NaCl]) for 2 h followed by overnight incubation with an anti-NS3 mouse polyclonal antibody or an anti-GAPDH rabbit polyclonal antibody (Abcam, USA) in blocking solution. The membrane was then washed three times with TBST and incubated with anti-mouse IgG or anti-rabbit IgG conjugated to horseradish peroxidase (Promega, USA) in blocking solution for 2 h. The membrane was washed again and developed with a SuperSignal West Pico kit (Pierce, IL, USA) and the image was acquired using an ImageQuant LAS-4000 (GE Healthcare, USA).

### Measurement of GAPDH glycolytic activity in NS3-transfected and DENV2-infected BHK-21 cells

The GAPDH activity was measured in a coupled reaction with triose phosphate isomerase (TPI) enzyme. Approximately 10^6^ mock- or DENV2-infected BHK-21 cells, or 10^6^ pcDNA3.1- or pcDNA3.1-NS3-transfected BHK-21 cells were lysed by adding 200 μL of IP-Lysis buffer (Pierce) followed by a 10 min ice-bath incubation and centrifugation at 17,900 *g* for 15 min. Fifty micrograms of the supernatant (cell extract) was added to the reaction mixture composed of: 90 mM triethanolamine, 1 mM EDTA pH 7.5, 3 mM potassium arsenate, 2 mM β-NAD^+^ and 2 U/mL of TPI (Sigma Aldrich). The reaction was started with the addition of 1 mM dihydroxyacetone phosphate (DHAP, Sigma Aldrich) and monitored by the change in absorbance at 340 nm every 5 min for 90 min. The absorbance units were converted into NADH production using the molar absorptivity of NADH of 6.22 mM^−1^ cm^−1^. One unit of GAPDH activity was defined as the reduction of 1 µM β-NAD^+^/min. The statistical analysis was carried out based on unpaired Student’s t test (Prism 7), obtaining p < 0.05.

### Immunofluorescence confocal microscopy

Huh7.5.1 cells were plated onto glass coverslips, infected with DENV2 strain 16681 for 48 h, washed in PBS buffer, pH 7.4, and fixed for 30 min with a freshly prepared 4% formaldehyde solution. The cells were permeabilized for 15 min with 0.6% saponin in PBS buffer, pH 7.4. The coverslips were incubated in blocking solution containing 1.5% BSA in PBS and then incubated for 1 h with purified anti-NS3 helicase polyclonal antibodies (made in-house) and anti-GAPDH polyclonal antibodies (Abcam, USA) diluted 1:100 in blocking solution. The cells were washed and incubated for 45 min with an Alexa 488-conjugated goat-anti-mouse IgG antibody or with an Alexa 546-conjugated goat anti-rabbit IgG antibody (Invitrogen, USA) diluted 1:400 in blocking solution. The cells were subsequently incubated with 5 µM 4′,6-diamidino-2-phenylindole (DAPI, Sigma, USA) for 10 min at room temperature. The slides were mounted in N-propyl gallate and observed using a Leica TCS SP5 confocal microscope. All images were collected with LAS AF Lite 2.6 software (Leica Microsystems, USA). The same protocol was used for the pcDNA3.1- and pcDNA3.1-NS3-transfected BHK-21 cells.

### Ethics statement

All procedures performed during this work were approved by the Ethics Committee of the Oswaldo Cruz Foundation/FIOCRUZ, under number 1.308.318 for studies with the fatal dengue case and controls. The institutional review board or ethics committee waived the need for consent.

#### Fatal human case

A 63-year-old male presented with a sudden onset of headache, myalgia, anorexia, abdominal pain, rash and hemorrhagic petechiae. Four days later, diarrhea and hemoptysis were observed, with leucopenia, hemoconcentration and thrombocytopenia confirmed by blood examination. This progressive deterioration led to the clinical picture of shock and acute pulmonary congestion, followed by DSS. The patient died with a clinical diagnosis of dengue hemorrhagic fever, acute heart failure and pancreatitis caused by DENV serotype 3 (DENV3).

### Histopathological and confocal immunofluorescence assay

Liver tissue samples from human necropsies were fixed with 10% formalin, blocked in paraffin resin, cut into 4-µm sections, deparaffinized in xylene and rehydrated with alcohol as described previously^12^. The sections were stained with hematoxylin and eosin for histological examination and visualized on a Nikon ECLIPSE E600 microscope.

Antigen retrieval was performed by heating the tissue in the presence of citrate buffer. Then, the tissues were blocked with 2% bovine serum albumin (BSA) for 40 min and permeabilized with 0.5% Triton X-100 for 10 min at room temperature. The slides were co-stained overnight at 4 °C with a purified mouse polyclonal anti-NS3 helicase antibody diluted 1:100 and a rabbit polyclonal anti-GAPDH antibody (Abcam) diluted 1:100 and left overnight at 4 °C. The sections were washed in PBS and incubated with an Alexa 488-conjugated rabbit anti-mouse IgG (Thermo, USA) and an Alexa 555-conjugated goat anti-rabbit IgG (Thermo, USA). The slides were mounted in Prolong Gold (Invitrogen, USA) and analyzed using a Zeiss LSM 510 Meta confocal microscope (Carl Zeiss, Oberkochen, Germany).

### Statistical analysis

Datasets were compared by a two-tailed, unpaired Student’s t-test, and statistical significance was set at p < 0.05. Multiple comparisons were performed using two-way ANOVA (Bonferroni post-test), and asterisks indicate significant differences with respect to the control (*p < 0.05, **p < 0.01, and ***p < 0.001).

## Supplementary information

Supplementary Information

## Data Availability

All data generated or analyzed during this study are included in this published article.
