# Supplementary material for: Dengue virus nonstructural 3 protein interacts directly with human glyceraldehyde-3-phosphate dehydrogenase (GAPDH) and reduces its glycolytic activity
Source: Sci Rep. 2019 Feb 25;9:2651. doi: 10.1038/s41598-019-39157-7 (PMC6389977; doi:10.1038/s41598-019-39157-7)
Supplement: Supplementary file 1 — Supplementary Information [file 41598_2019_39157_MOESM1_ESM.pdf]

Dengue virus nonstructural 3 protein interacts directly with human glyceraldehyde-3-phosphate dehydrogenase (GAPDH) and reduces its glycolytic activity

Emiliana M. Silva[1], Jonas N. Conde[1], Diego Allonso[2], Gustavo T. Ventura[1], Diego R. Coelho[1], Pedro Henrique Carneiro[1], Manuela L. Silva[3], Marciano V. Paes[4], Kíssila Rabelo[5], Gilberto Weissmuller[6], Paulo Mascarello Bisch[6], Ronaldo Mohana-Borges[1*].

[1]Laboratório de Genômica Estrutural, Instituto de Biofísica Carlos Chagas Filho, Universidade Federal do Rio de Janeiro, Rio de Janeiro, RJ 21941-590, Brazil.

[2]Departamento de Biotecnologia Farmacêutica, Faculdade de Farmácia, Universidade Federal do Rio de Janeiro, Rio de Janeiro, RJ, Brazil.

[3]Instituto de Biodiversidade e Sustentabilidade (NUPEM/UFRJ), Universidade Federal do Rio de Janeiro, Macaé, RJ, Brazil.

[4]Laboratório Interdisciplinar de Pesquisa Médica, Instituto Oswaldo Cruz, Fundação Oswaldo Cruz, Rio de Janeiro, RJ, Brazil.

[5]Laboratório de Ultraestrutura e Biologia Tecidual, Universidade Estadual do Rio de Janeiro, Rio de Janeiro, RJ, Brazil.

[6]Laboratório de Física Biológica, Instituto de Biofísica Carlos Chagas Filho, Universidade Federal do Rio de Janeiro, Rio de Janeiro, RJ 21941-590, Brazil.

[*]Corresponding author: Ronaldo Mohana-Borges, Ph.D., Laboratório de Genômica Estrutural

Supplemental Table S1 - Amino acid residues found at the protein interface between the full-length DENV2 NS3 (protease-helicase domains) and GAPDH proteins

NS3 protease domain		NS3 helicase domain	GAPDH	
ALA16	LYS142	ILE178	ASP81	TRP196
GLU17	LYS143	ASP180	VAL101	LYS215
LEU18	GLY144	ASP181	LYS107	GLY226
GLU19	LYS145	ARG184	VAL171	LYS227
ASP20		PRO205	GLU172	THR229
LYS42		ALA206	LEU174	GLY230
GLU43		ARG209	THR176	MET231
LYS84		ILE212	PRO191	PHE233
GLU86		LYS213	SER192	THR246
GLN96		ALA234	GLY193	ARG248
ILE140			LYS194	LYS309

Note: The residues above are represented as spheres in Figures 5A and 5D.

Supplemental Table S2 - Amino acid residues found at the protein interface between the helicase DENV2 NS3 and GAPDH proteins

NS3 helicase domain		GAPDH	
MET306	ARG524	GLU172	THR229
GLY437	LEU525	LEU174	MET231
GLU438	ARG526	THR176	PHE233
PRO501	GLU528	ASP189	ASP244
GLU502	ALA529	TRP196	THR246
GLY503	TYR548	ARG197	ARG248
ILE504	ARG549	ARG200	ASN304
ILE505	ALA522	ASN205	HIS306
ILE519	GLU553	ILE206	PHE307
ASP520	GLY554	PRO208	
GLY521	ILE555	SER210	
GLU522	ARG594	LYS215	

Note: The residues above are represented as spheres in Figures 5B and 5E.

Supplemental Table S3 - Amino acid residues found at the protein interface between the protease denv2 ns3 and gapdh proteins

NS3 protease domain	GAPDH
ALA16	PRO191
GLU17	SER192
LEU18	TRP196
GLU19	ARG197
ASP20	ARG200
GLU43	PRO208

Note: The residues above are represented as spheres in Figures 5C and 5F.

Supplemental Table S4 - Amino acid residues found at the protein interface between the DENV2 NS1 and GAPDH proteins

GAPDH	
VAL171	ASP244
GLU172	THR246
LEU174	ARG248
THR176	TYR255
VAL178	GLY298
LEU195	ALA299
TRP196	ILE301
ARG197	LEU303
ARG200	ASN304
PRO208	ASP305
THR229	HIS306
MET231	PHE307
PHE233	LYS309
VAL242	TRP313

Note: The residues above are represented as spheres in the Figs. 5G.
Only amino acid residues of the GAPDH protein is shown.

Supplemental Figure S1 – Detection of intracellular and secreted NS1 and NS3 proteins in DENV2-infected cells

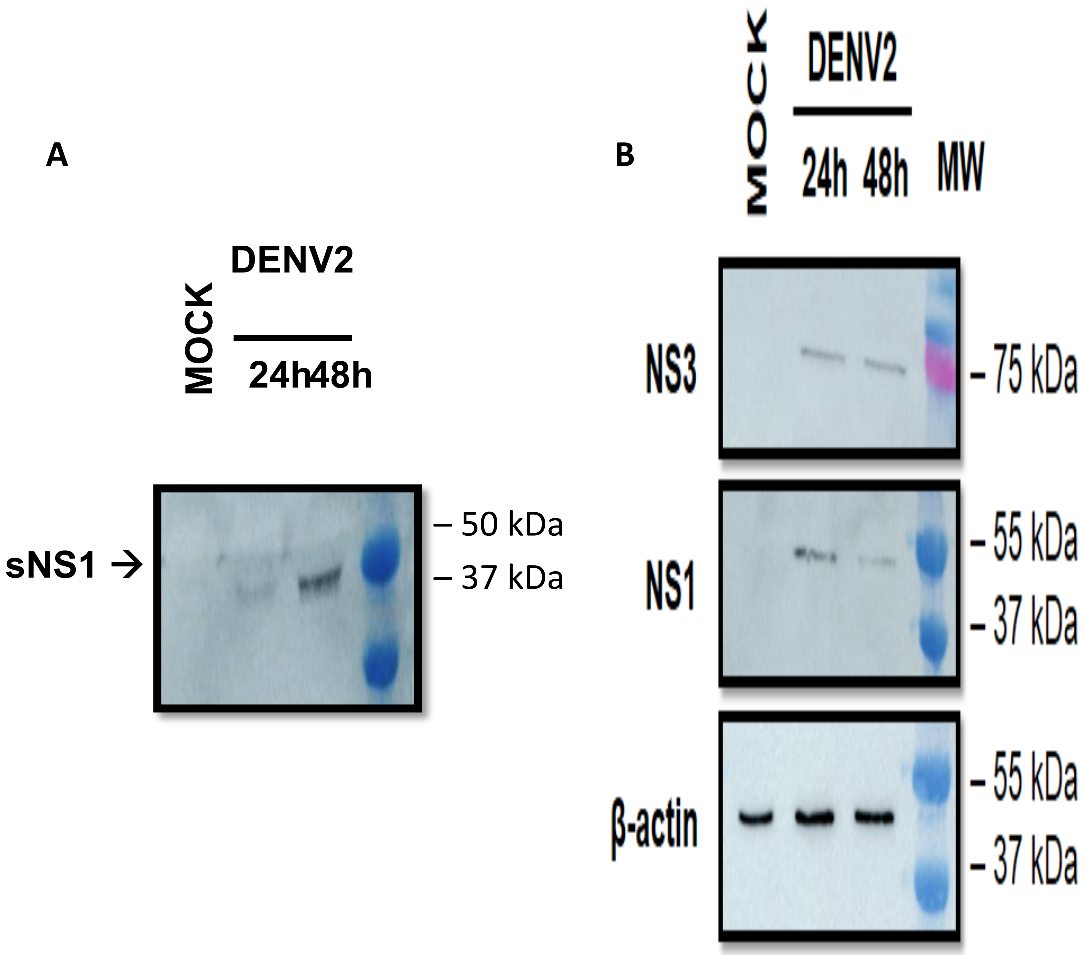

(A) Supernatant from mock or DENV-infected BHK-21 cells analyzed by Western blot with anti-NS1 antibody. Bands of 50 kDa were observed corresponding to secreted NS1 protein. (B) Cellular extract of mock or DENV-infected BHK-21 cells analyzed by Western blot with anti-NS3, anti-NS1. Bands of 70 and 50 kDa were observed corresponding to intracellular NS3 and NS1 proteins. Anti-β-actin was used as loading control.

Method: BHK-21 cells seeded in 24-well plate were mock or infected with DENV2 (MOI=1). In 24 and 48 h post-infection, the culture supernatant was collected and analyzed by Western blot anti-NS1 antibody. Cell extracts were harvested with 200 μL RIPA buffer, quantified by Bradford assay and 6 μg were analyzed by Western blot using anti-DENV-NS1, anti-DENV-NS3 and anti- β-actin antibodies.

Supplemental Figure S2 – Detection of intracellular and secreted NS1 and NS3 proteins in DENV2-infected cells (whole gels of Supplemental Fig. S1).

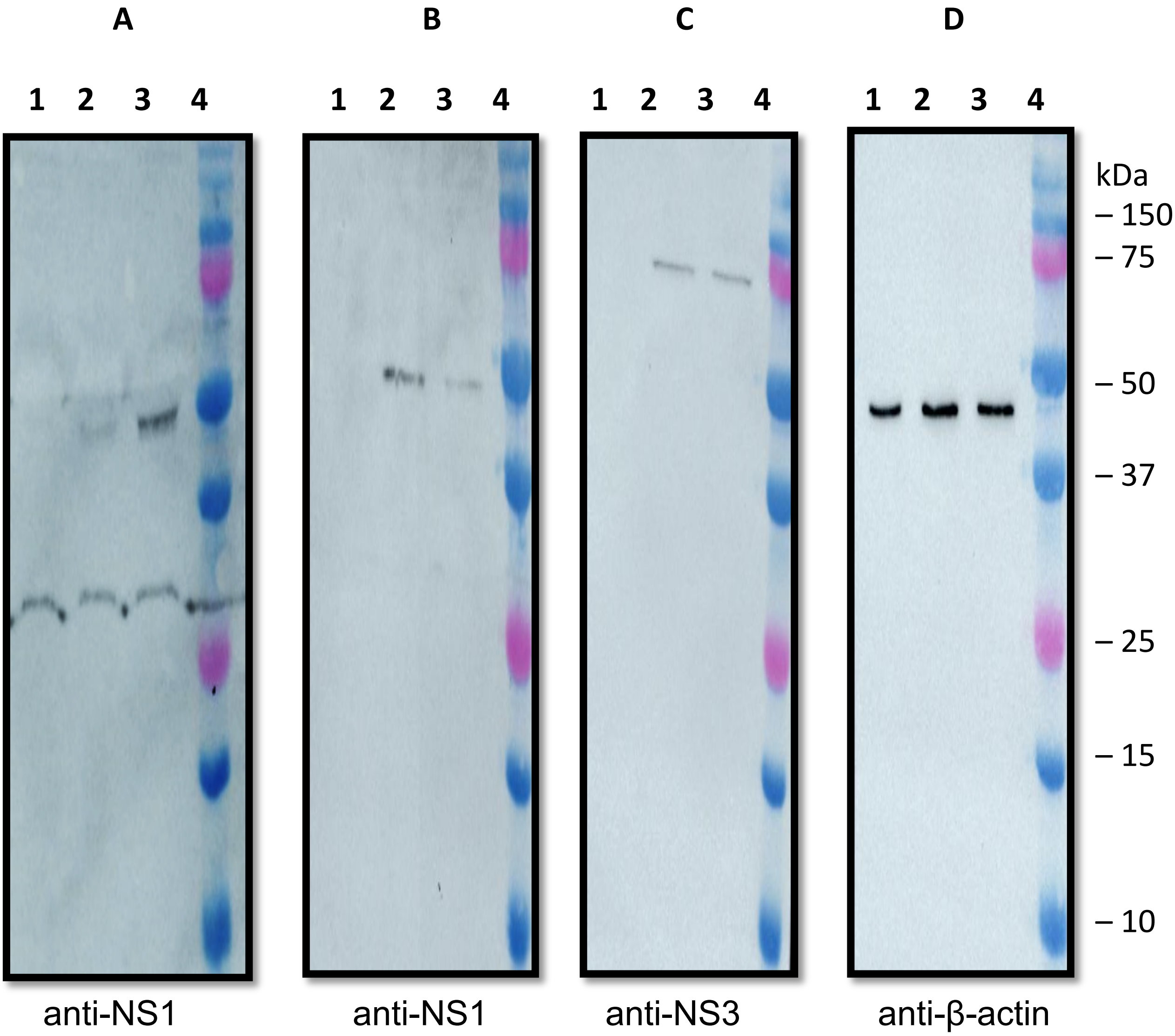

Supernatant (A) and intracellular extract (B, C and D) from mock (lane 1) or DENV-infected BHK-21 cells at 24 h (lane 2) and 48 h (lane 3) were analyzed by Western blot. Bands of 50 kDa were observed corresponding to secreted (A) and intracellular (B) NS1 protein with anti-NS1 antibody. (C) Cellular extract of mock or DENV-infected BHK-21 cells analyzed by Western blot with anti-NS3 antibody. Bands of 70 kDa were observed corresponding to intracellular NS3 protein. (D) Cell extract of mock or DENV-infected BHK-21 cells analyzed by Western blot with anti-β-actin antibody as loading control. Bands of 45 kDa were observed corresponding to β-actin.

Supplemental Figure S3 - Co-IP with IgG (negative control) antibody coupled to the column (duplicate 1)

Western blotting revealed with rabbit anti-GAPDH antibody from Abcam (dilution 1:500) (whole gel of Figure 6B)

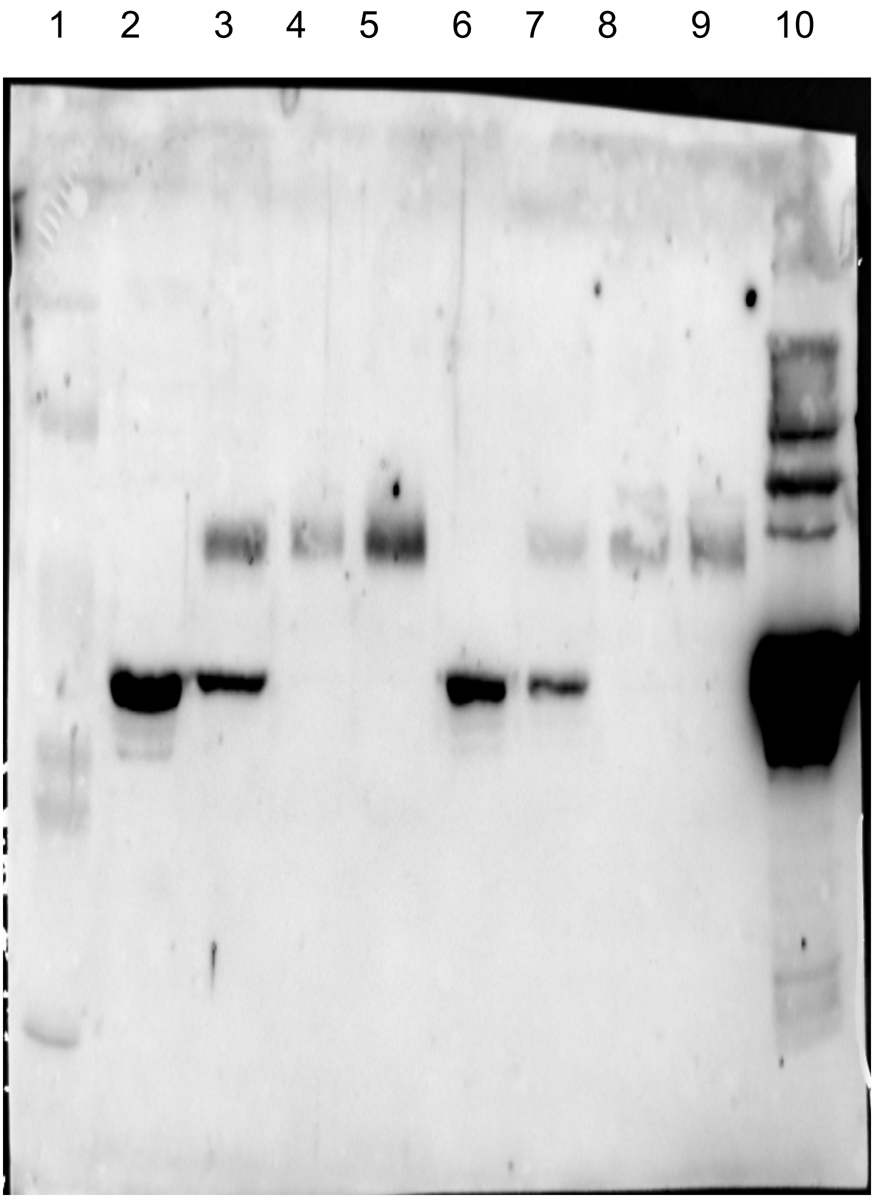

Sample order in the gel lanes:

1. Molecular Weight
2. Input of DENV2-infected cells at 48h
3. Flow through of DENV2 Co-IP
4. Elution 1 DENV2 Co-IP
5. Elution 2 DENV2 Co-IP
6. Input DENV2-infected cells 48h
7. Flow through DENV2 Co-IP
8. Elution 1 DENV2 Co-IP
9. Elution 2 DENV2 Co-IP
10. Purified GAPDH (Abcam)

Supplemental Figure S4 - Co-IP with IgG (negative control) antibody coupled to the column (duplicate 1)

Western blotting revealed with rabbit anti-NS3 antibody previously purified in protein G column (dilution 1:500) (whole gel of Figure 6B)

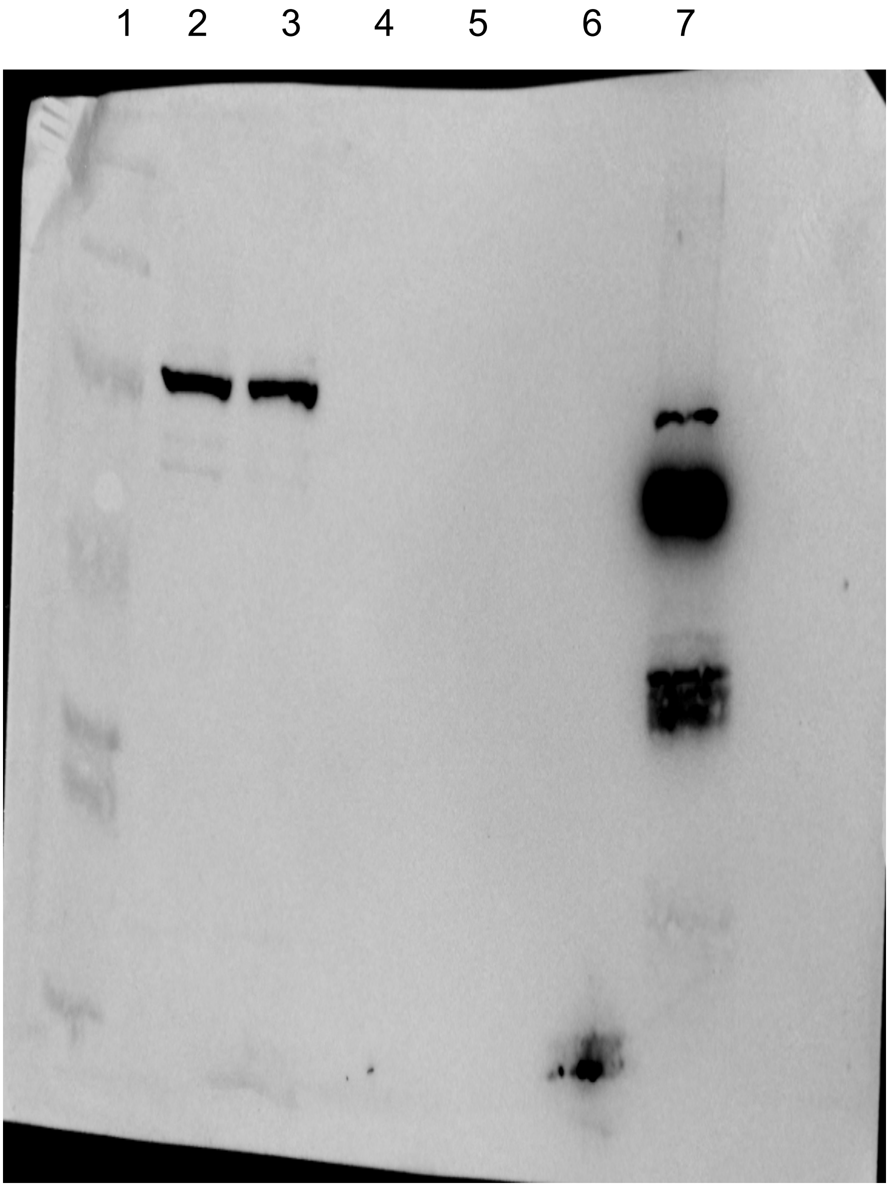

Sample order in the gel lanes:

1. Molecular Weight
2. Input DENV2-infected cell extract
3. Flow through DENV2 Co-IP IgG
4. Elution 1 Co-IP IgG
5. Elution 2 Co-IP IgG
6. Purified GAPDH
7. Purified Full-length DENV2 NS3

Supplemental Figure S5 - Co-IP with rabbit anti-GAPDH antibody (Abcam) coupled to the column

Western blotting revealed with rabbit purified anti-NS3 antibody (dilution 1:200) and blocked with 5% BSA for 1 hour (whole gel of Figure 6A)

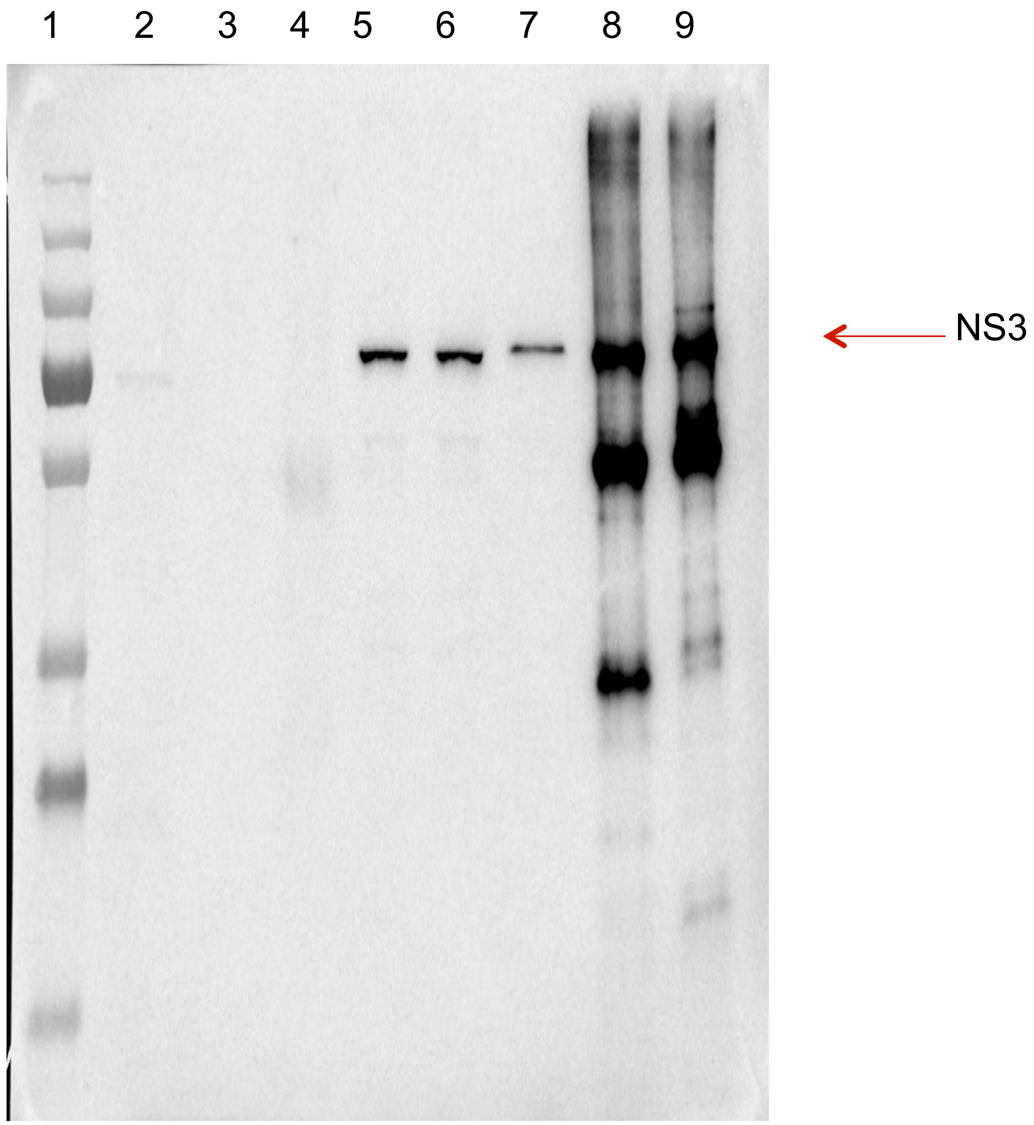

Sample order in the gel lanes:

1. Molecular Weight
2. Input Mock-infected cells 48h
3. Flow through Mock Co-IP
4. Elution Mock Co-IP
5. Input DENV2-infected cells MOI=2
6. Flow through DENV2 co-IP
7. Elutions DENV2 Co-IP
8. Purified Full-length NS3
9. Purified Full-length NS3 (enz. assay)

Supplemental Figure S6 - Co-IP with rabbit anti-GAPDH antibody (Abcam) coupled to the column

Western blotting revealed with rabbit anti-GAPDH antibody from Abcam (dilution 1:200) and blocked with 2% BSA for 1 hour (whole gel of Figure 6A)

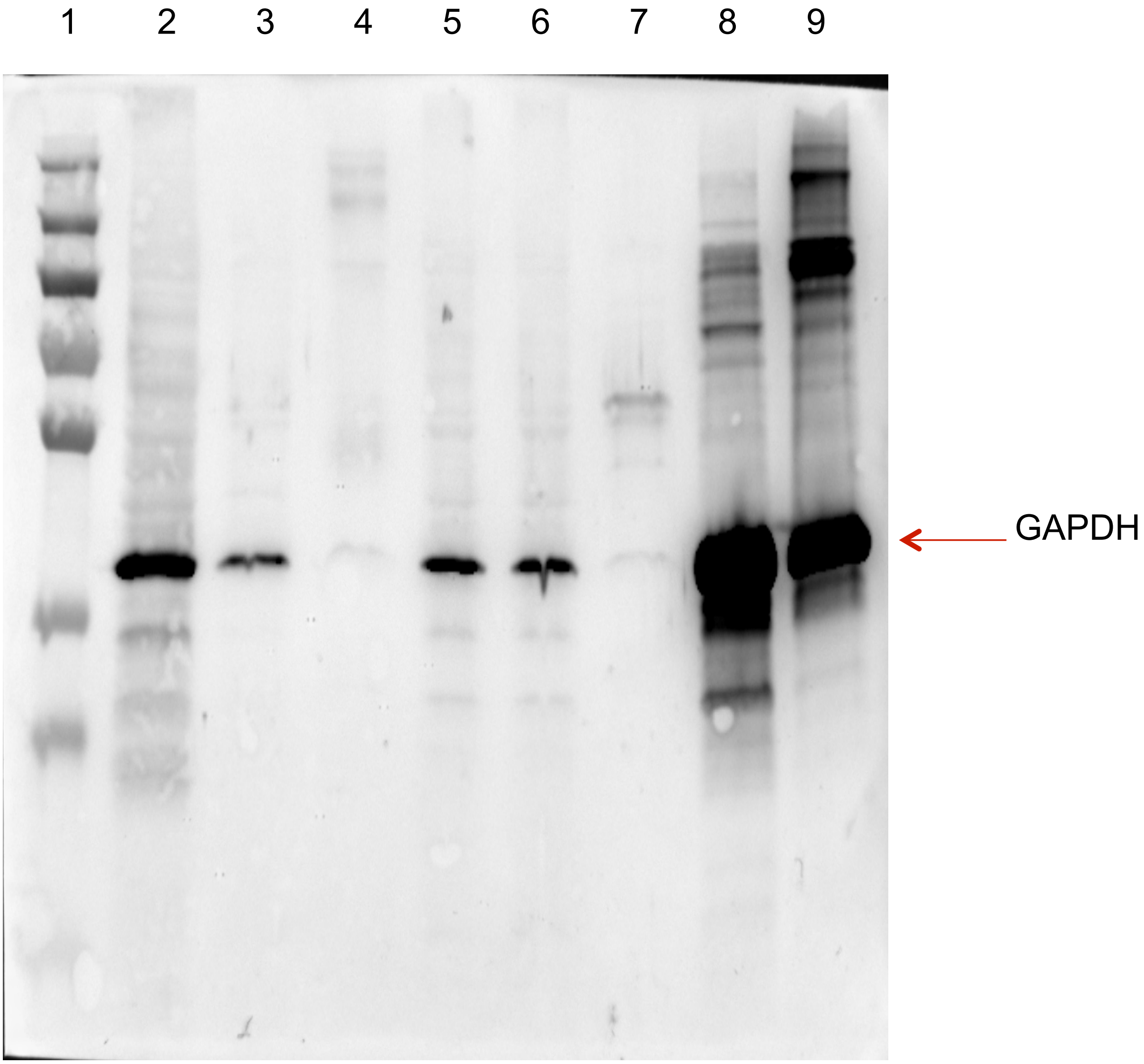

Sample order in the gel lanes:

1. Molecular Weight
2. Input Mock-infected cells 48h
3. Flow through Mock Co-IP
4. Elution Mock Co-IP
5. Input DENV2-infected cell (MOI=2)
6. Flow through DENV2 co-IP
7. Elution DENV2 Co-IP
8. Purified GAPDH (Abcam) 10 µg
9. Purified GAPDH (Abcam) 5 µg

Supplemental Figure S7 - Co-IP with rabbit anti-GAPDH antibody (Abcam) coupled to the column

Western blotting revealed with rabbit anti-GAPDH antibody from Abcam (dilution 1:200) and blocked with 2% BSA for 1 hour (whole gel of Figure 6A)

Same Figure as in Supplemental Figure S6 but with 1 min exposure

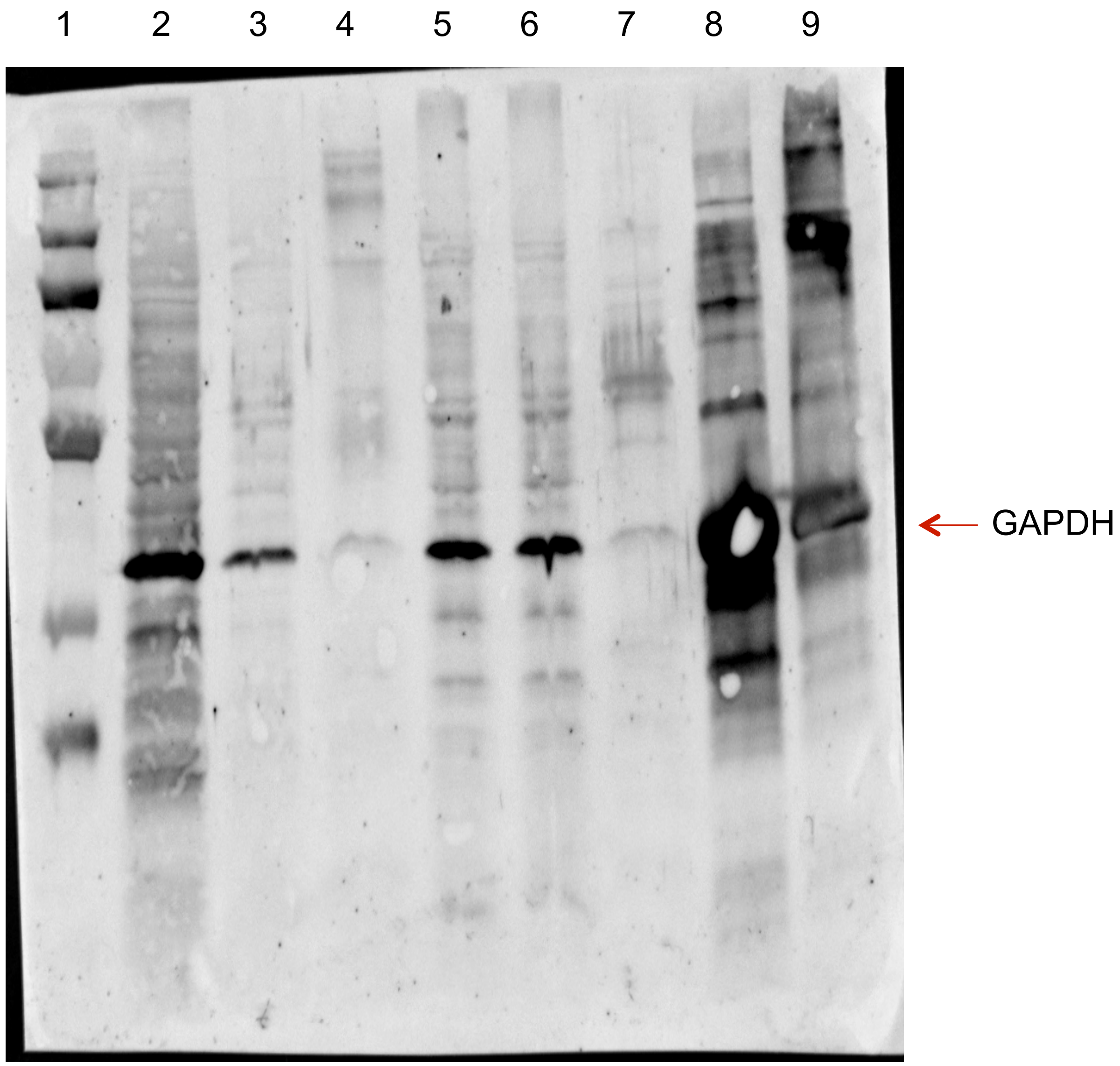

Sample order in the gel lanes:

1. Molecular Weight
2. Input Mock-infected cells 48h
3. Flow through Mock Co-IP
4. Elution Mock Co-IP
5. Input DENV2-infected cell (MOI=2)
6. Flow through DENV2 co-IP
7. Elution DENV2 Co-IP
8. Purified GAPDH (Abcam) 10 µg
9. Purified GAPDH (Abcam) 5 µg

Supplemental Figure S8 - Co-IP with rabbit anti-NS3 antibody coupled to the column

Western blotting revealed with rabbit anti-GAPDH antibody from Abcam (dilution 1:2000) and blocked with 5% BSA for 1 hour (whole gel of Figure 6C)

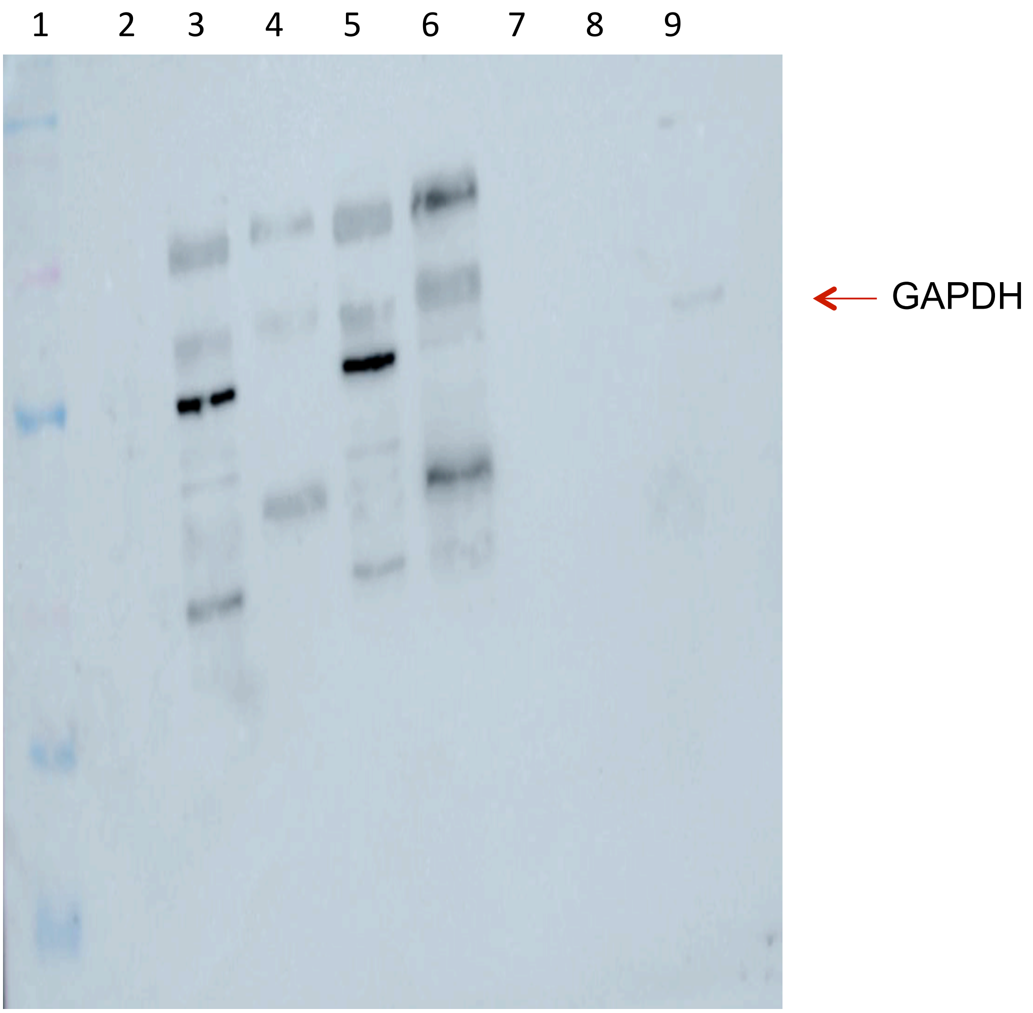

Sample order in the gel lanes:

1. Molecular Weight
2. Empty lane
3. Input Co-IP Control
4. Elution Co-IP Control
5. Input Co-IP NS3 transfected
6. Elution Co-IP NS3 transfected
7. Empty lane
8. Purified Full-length NS3 protein
9. Purified GAPDH (Abcam)

Supplemental Figure S9 - Co-IP with rabbit anti-NS3 antibody coupled to the column

Western blotting revealed with mouse anti-GAPDH antibody (dilution 1:2000) and blocked with 5% BSA for 1 hour (whole gel of Figure 6C)

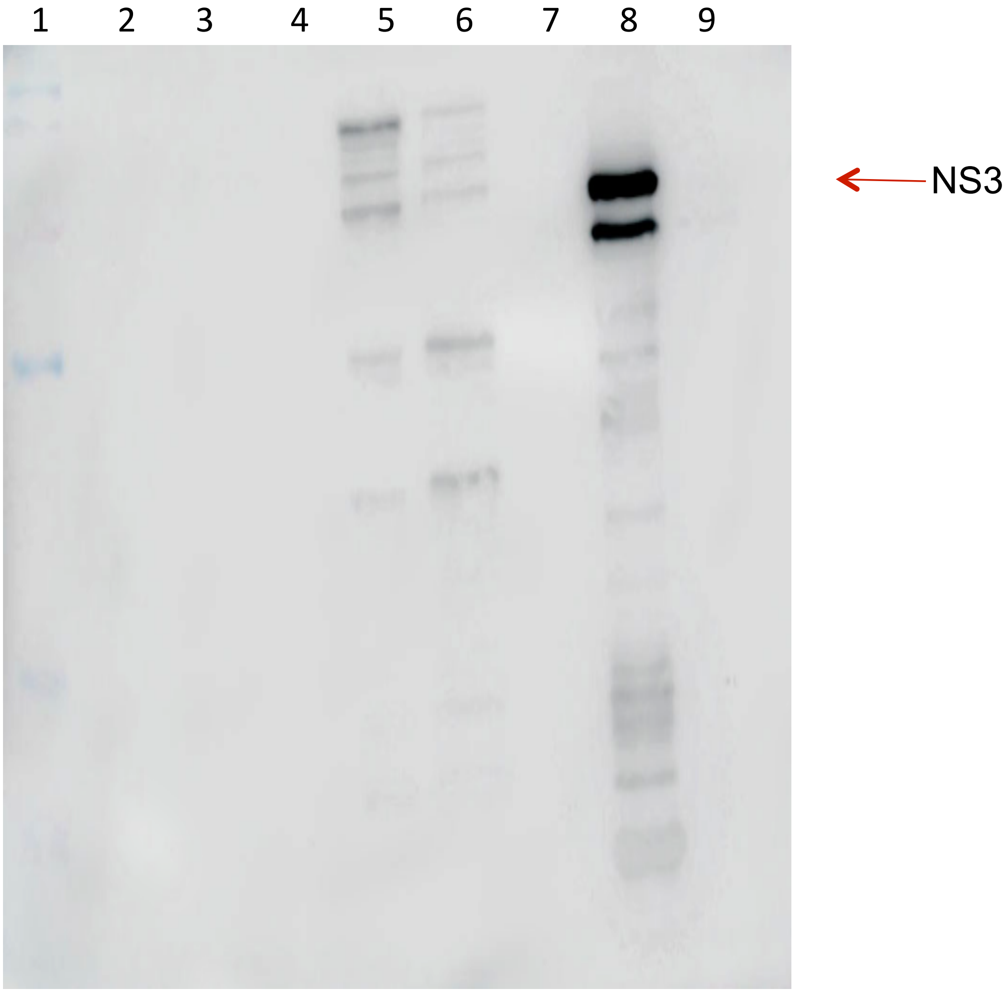

Sample order in the gel lanes:

1. Molecular Weight
2. Empty lane
3. Input Co-IP Control
4. Elution Co-IP Control
5. Input Co-IP NS3 transfected
6. Elution Co-IP NS3 transfected
7. Empty lane
8. Purified Full-length NS3 protein
9. Purified GAPDH (Abcam)

Supplemental Figure S9 – Amino acid sequences of the full-length DENV2 NS3 (New Guinea Strain) and human GAPDH proteins

NS3 protein

AGVLWDVPSPPPVGKAELEDGAYRIKQKGILGYSQIGAGVYKEGTFHTMWHVTRGAVLMHKGKRI
EPSWADVKKDLISYGGGWKLEGEWKEGEEVQVLALEPGKNPRAVQTKPGLFKTNAGTIGAVSLDF
SPGTSGSPIIDKKGKVVGLYGNGVVTRSGAYVSAIAQTEKSIEDNPEIEDDIFRKRKLTIMDLHPGAGK
TKRYLPAIVREAIKRGLRTLILAPTRVVAAEMEEALRGLPIRYQTPAIRAEHTGREIVDLMCHATFTMR
LLSPVRVPNYNLIIMDEAHFTDPASIAARGYISTRVEMGEAAGIFMTATPPGSRDPFPQSNAPIMDE
EREIPERSWSSGHEWVTDFKGKTVWFVPSIKAGNDIAACLRKNGKKVIQLSRKTFDSEYVKTRTND
WDFVVTTDISEMGANFKAERVIDPRRCMKPVILTDGEERVILAGPMPVTHSSAAQRRGRIGRNPK
NENDQYIYMGEPLENDEDCAHWKEAKMLLDNINTPEGIIPSMFEPEREKVDAIDGEYRLRGEARKT
FVDLMRRGDLPVWLAYRVAAEGINYADRRWCFDGIKNNQILEENVEVEIWTKEGERKKLKPRWLD
ARI YSDPLALKEFKEFAAGRK

Note: the blue and black sequences correspond to the protease and helicase domains respectively.

Human GAPDH protein

GSHMGKVKVGVNGFGRIGRLVTRAAFNSGKVDIVAINDPFIDLNYMVYMFQYDSTHGKFHGTVKA
ENGKLVINGNPITIFQERDPSKIKWGDAGAEYVVESTGVFTTMEKAGAHLQGGAKRVIISAPSADAP
MFVMGVNHEKYDNSLKIISNASCTTNCLAPLAKVIHDNFGIVEGLMTTVHAITATQKTVDGPSGKL
WRDGRGALQNIIPASTGAAKAVGKVIPELNGKLTGMAFRVPTANVSVVDLTCRLEKPAKYDDIKKV
VKQASEGPLKGILGYTEHQVVSSDFNSDTHSSTFDAGAGIALNDHFVKLISWYDNEFGYSNRVVDL
MAHMASKE
